# Supplementary material for: Zinc-indium-sulfide favors efficient C − H bond activation by concerted proton-coupled electron transfer
Source: Nat Commun. 2024 Jun 11;15:4967. doi: 10.1038/s41467-024-49265-2 (PMC11167015; doi:10.1038/s41467-024-49265-2)
Supplement: Supplementary file 1 — Supplementary Information [file 41467_2024_49265_MOESM1_ESM.pdf]

Supplemental Methods

General considerations

Products from photocatalytic coupling reactions were identified by an Agilent HP 6890N gas chromatograph (GC) equipped with an HP5-MS capillary column and an Agilent 5973 mass-spectrometer detector. These coupling products were quantified by an Agilent HP 6890N GC equipped with an HP-5 capillary column and a flame ionization detector (FID). For the coupling of toluene, the yields of products were calibrated by using commercial chemicals as standards. For the transformation of other chemicals, the conversion was calculated using the following equation: 100% $\times$ (1-$n_{remaining\ reactant}/n_{initial\ reactant}$); the yields of products were calculated by assuming these products have similar FID responsive factor as the reactants. GC-FID operation conditions are as followings: injection temperature, 300 °C; column temperature program for most coupling products: 100 °C for 3 min, increasing the temperature to 300 °C at a rate of 10 °C min^{-1}, holding at 300 °C for 5 min; column temperature program for products from the coupling of phenols and benzyl alcohols, which are with relatively high boiling points: 50 °C for 1 min, increasing the temperature to 150 °C at a rate of 15 °C min^{-1}, increasing the temperature from 150 °C to 300 °C at a rate of 10 °C min^{-1}, holding at 300 °C for 18 min; detection temperature, 300 °C; split ratio: 25. The injection temperature and column temperature program for GC-FID was also adopted for GC-MS. The solvent retention time was 3 minutes for GC-MS. H_2 was measured by an INFICON Micro GC Fusion equipped with a molecular sieve 5A column and a high-sensitivity thermal conductivity detector.

Semiconductors in this study were characterized by powder X-ray diffraction (XRD), transmission electron microscopy (TEM), diffuse reflectance spectroscopy (DRS), and photoelectrochemical measurements. XRD patterns were recorded on a high-throughput STOE STADI P Combi diffractometer using Cu K_α radiation. TEM and high-resolution TEM (HRTEM) measurements were performed on a Tecnai F20 electron microscope (Phillips Analytical) operated at an acceleration voltage of 200 kV. DRS of as-prepared samples was measured with an ultraviolet-visible spectrophotometer (UV–vis, Lambda-950). Photoelectrochemical measurements were carried out using a standard three-electrode cell with a working electrode, and calomel (Hg/Hg_2Cl_2) reference electrode, and a Pt plate as the counter electrode. A 0.1 M Na_2SO_4 aqueous solution was used as the electrolyte. The working electrode was prepared based on an F-doped SnO_2-coated glass (FTO glass, 0.5 cm × 0.5 cm). The photocatalyst was dispersed in acetone, which contains 1% Nafion D521 dispersion, by ultrasonic treatment, and the suspension was added dropwise onto the FTO by microsyringe. The surface density of the photocatalysts was 1 mg cm^{-2}. The film was dried in the air overnight. The potential and the frequency range of the Mott-Schottky measurements are from -0.8 to 0.8 V (*vs.* SCE) and from 500 to 1500 Hz, respectively.

Derivation of rate expressions

<u>For CPET C–H activation</u>

<u>Possibility 1:</u>

When A2 in Scheme 1 is the rate-determining step:

Adsorption of RC–H: $k_{Ad1} \times c_{RC–H} \times \Theta_{\{[AS]\}} - k_{De1} \times \Theta_{\{RC–H[S]\}} = 0$

$$\rightarrow \Theta_{\{RC–H[S]\}} = K_{Ad1} \times c_{RC–H} \times \Theta_{\{[AS]\}},\ K_{Ad1} = k_{Ad1}/k_{De1} \quad (a1)$$

Adsorption of RC•: $k_{De2} \times \Theta_{\{RC•[S]\}} - k_{Ad2} \times c_{RC•} \times \Theta_{\{[AS]\}} = 0$

$$\rightarrow \Theta_{\{RC•[S]\}} = K_{Ad2} \times c_{RC•} \times \Theta_{\{[AS]\}},\ K_{Ad2} = k_{Ad2}/k_{De2} \quad (a2)$$

$$1 = \Theta_{\{RC–H[S]\}} + \Theta_{\{RC•[S]\}} + \Theta_{\{[AS]\}} \quad (a3)$$

Substitute (a1) and (a2) into (a3)

$$\Theta_{\{[AS]\}} = \frac{1}{K_{Ad1} \times c_{RC–H} + K_{Ad2} \times c_{RC•} + 1} \quad (a4)$$

$$r_{CPET} = k_{CPET} \times \Theta_{\{RC–H[S]\}} \times h_s^+ \quad (a5)$$

Substitute (a1) (a4) into (a5)

$$r_{CPET} = k_{CPET} \times \frac{K_{Ad1} \times c_{RC–H}}{K_{Ad1} \times c_{RC–H} + K_{Ad2} \times c_{RC•} + 1} \times h_s^+ \quad (a6)$$

Since $•PhCH_2$ undergoes quick C–C coupling reaction, the coupling product generation rate by CPET mechanism over Zn-In-S ($r_{Zn\text{-}In\text{-}S}$) can be described by the following expression:

$$r_{Zn\text{-}In\text{-}S} = \frac{1}{2} \times r_{CPET} = k_{CPET} \times \frac{K_{Ad1} \times c_{RC–H}}{2 \times (K_{Ad1} \times c_{RC–H} + K_{Ad2} \times c_{RC•} + 1)} \times h_s^+ \quad (a7)$$

<u>Possibility 2:</u>

When A4 in Scheme 1 is the rate-determining step, the above (a1), (a2), (a3), and (a4) are still validated, while the above (a5), (a6), and (a7) are no longer validated.

Instead, for the generation of H^+ and RC•[S]:

$k_{CPET} \times \Theta_{\{RC–H[S]\}} \times h_s^+ - k_{-CPET} \times \Theta_{\{RC•[S]\}} \times c_{H+} = 0$

$$\rightarrow c_{H+} = K_{CPET} \times \Theta_{\{RC–H[S]\}} \times h_s^+ \div \Theta_{\{RC•[S]\}},\ K_{CPET} = k_{CPET}/k_{-CPET} \quad (a8)$$

The coupling product generation rate over Zn-In-S ($r_{Zn\text{-}In\text{-}S}$) equals to the rate for hydrogen generation (r_{H2}):

$$r_{Zn\text{-}In\text{-}S} = r_{H2} = k_R \times c_{H+}^2 \times e_s^{-2} \quad (a9)$$

Substitute (a1) (a2), (a4), and (a8) into (a9):

$$r_{\text{Zn-In-S}} = k_R \times \frac{(K_{\text{CPET}} \times K_{\text{Ad1}} \times c_{\text{RC–H}})^2}{(K_{\text{Ad2}} \times c_{\text{RC}\cdot})^2} \times (h_s^+ \times e_s^-)^2 \qquad \text{(a10)}$$

For ET-PT C−H activation

Possibility 1:

When B2 in Scheme 1 is the rate-determining step:

Adsorption of RC−H: $k_{\text{Ad1}} \times c_{\text{RC–H}} \times \Theta_{\{[\text{AS}]\}} - k_{\text{De1}} \times \Theta_{\{\text{RC–H[S]}\}} = 0$

$$\rightarrow \Theta_{\{\text{RC–H[S]}\}} = K_{\text{Ad1}} \times c_{\text{RC–H}} \times \Theta_{\{[\text{AS}]\}},\ K_{\text{Ad1}} = k_{\text{Ad1}}/k_{\text{De1}} \qquad \text{(b1)}$$

Adsorption of RC−H$^+$: $k_{\text{De3}} \times \Theta_{\{\text{RC–H}^+[\text{S}]\}} - k_{\text{Ad3}} \times c_{\text{RC–H}^+} \times \Theta_{\{[\text{AS}]\}} = 0$

$$\rightarrow \Theta_{\{\text{RC–H}^+[\text{S}]\}} = K_{\text{Ad3}} \times c_{\text{RC–H}^+} \times \Theta_{\{[\text{AS}]\}},\ K_{\text{Ad3}} = k_{\text{Ad3}}/k_{\text{De3}} \qquad \text{(b2)}$$

$$1 = \Theta_{\{\text{RC–H[S]}\}} + \Theta_{\{\text{RC–H}^+[\text{S}]\}} + \Theta_{\{[\text{AS}]\}} \qquad \text{(b3)}$$

Substitute (b1) and (b2) into (b3)

$$\Theta_{\{[\text{AS}]\}} = \frac{1}{K_{\text{Ad1}} \times c_{\text{RC–H}} + K_{\text{Ad3}} \times c_{\text{RC–H}^+} + 1} \qquad \text{(b4)}$$

Because the deprotonation and C−C coupling reaction are both quick reaction, the coupling product generation rate by ET-PT mechanism over TiO_2 (r_{TiO2}) is:

$$r_{\text{TiO2}} = \frac{1}{2} \times r_{\text{RC–H+}} = \frac{1}{2} \times k_{\text{ET}} \times \Theta_{\{\text{RC–H[S]}\}} \times h_s^+ \qquad \text{(b5)}$$

Substitute (b1) and (b4) into (b5)

$$r_{\text{TiO2}} = k_{\text{ET}} \times \frac{K_{\text{Ad1}} \times c_{\text{RC–H}}}{2 \times (K_{\text{Ad1}} \times c_{\text{RC–H}} + K_{\text{Ad3}} \times c_{\text{RC–H}^+} + 1)} \times h_s^+ \qquad \text{(b6)}$$

Possibility 2

When B3 in Scheme 1 is the rate-determining step, the above (b1) and (b3) are still validated, while the above (b2), (b3), (b5), and (a6) are no longer validated.

ET reaction: $k_{\text{ET}} \times \Theta_{\{\text{RC–H[S]}\}} \times h_s^+ - k_{\text{-ET}} \times \Theta_{\{\text{RC–H}^+[\text{S}]\}} = 0$

$$\rightarrow \Theta_{\{\text{RC–H}^+[\text{S}]\}} = K_{\text{ET}} \times h_s^+ \times \Theta_{\{\text{RC–H[S]}\}} = K_{\text{ET}} \times h_s^+ \times K_{\text{Ad1}} \times c_{\text{RC–H}} \times \Theta_{\{[\text{AS}]\}} \qquad \text{(b7)}$$

$$\Theta_{\{[\text{AS}]\}} = \frac{1}{K_{\text{Ad1}} \times c_{\text{RC–H}} + K_{\text{ET}} \times h_s^+ \times K_{\text{Ad1}} \times c_{\text{RC–H}} + 1} \qquad \text{(b8)}$$

$$r_{\text{TiO2}} = \frac{1}{2} \times r_{\text{RC–H+}} = \frac{1}{2} \times k_{\text{De3}} \times \Theta_{\{\text{RC–H}^+[\text{S}]\}} \qquad \text{(b9)}$$

$$r_{TiO2} = k_{De3} \times \frac{K_{ET} \times K_{Ad1} \times c_{RC-H}}{2 \times (K_{Ad1} \times c_{RC-H} + K_{ET} \times h_s^+ \times K_{Ad1} \times c_{RC-H} + 1)} \times h_s^+ \quad \text{(b10)}$$

Computational setup and methods

The spin polarized density functional theory (DFT) calculations were performed by using the projector augmented wave method[1] as implemented in the Vienna Ab initio Simulation Package[2,3]. The Grimme's D3 corrections[4] were used to describe the Van der Waals interactions. The Perdew−Burke−Ernzerhof (PBE) density functional[5] was employed. The plane wave cutoff of 500 eV was used. The Brillouin-zone integrations were approximated by using the k-point sampling of the Monkhorst−Pack scheme. The wave function optimization was considered to be converged when the energy change between two steps was smaller than 10^{-4} eV. The structures were optimized using the RMM-DIIS algorithm, and the convergence conditions for the ionic relaxation loop were that the forces on each ion were less than 0.05 eV/Å. The bulk lattice constants were optimized with 11 × 11 × 11 k-point meshes. The optimized lattice constants are a = 3.803 and c = 9.573 for anatase TiO_2, a = 3.888 and c = 12.412 for hexagonal $ZnIn_2S_4$, a = 3.875 and c = 30.703 for hexagonal $Zn_2In_2S_5$, which are in good agreement with the corresponding experimental values. The TiO_2 (101) surface models were represented by 3 × 3 slabs of 4 layers separated by 20 Å of vacuum in periodic cells. Surface calculations were done with 3 × 3 × 1 k-point meshes. The top two layers together with the adsorbates were allowed to fully relax, while the bottom two layers were fixed with dipole correction applied in z direction during optimization for all surface calculations. The $ZnIn_2S_4$(001) surface models were represented by 3 × 3 slabs of 2 layers separated by 30 Å of vacuum in periodic cells. Surface calculations were done with 3 × 3 × 1 k-point meshes. The top layer together with the adsorbates were allowed to fully relax, while the bottom layer was fixed with dipole correction applied in z direction during optimization for all surface calculations. The $Zn_2In_2S_5$(001) surface models were represented by 3 × 3 slabs of 1 layer separated by 30 Å of vacuum in periodic cells. Surface calculations were done with 3 × 3 × 1 k-point meshes. The top two atomic layers together with the adsorbates were allowed to fully relax, while the bottom two atomic layers were fixed with dipole correction applied in z direction during optimization for all surface calculations.

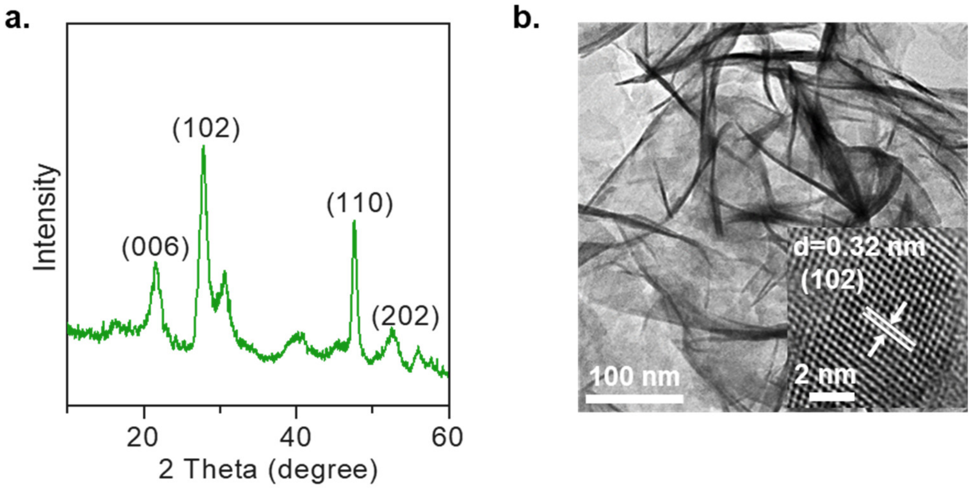

Supplementary Fig. 1 | Basic characterizations of synthesized $ZnIn_2S_4$. **a,** XRD patterns. **b,** TEM and HRTEM (inset) micrographs.

Supplementary Table 1 | BET surface area of Zn-In-S and TiO_2 photocatalysts in this study

Catalysts	BET surface area (m^2/g)		
Zn-In-S	$Zn_{0.5}In_2S_{3.5}$	$ZnIn_2S_4$	$Zn_2In_2S_5$
	95	62	58
TiO_2	A-{001}	P25	R-{110}
	43	52	12

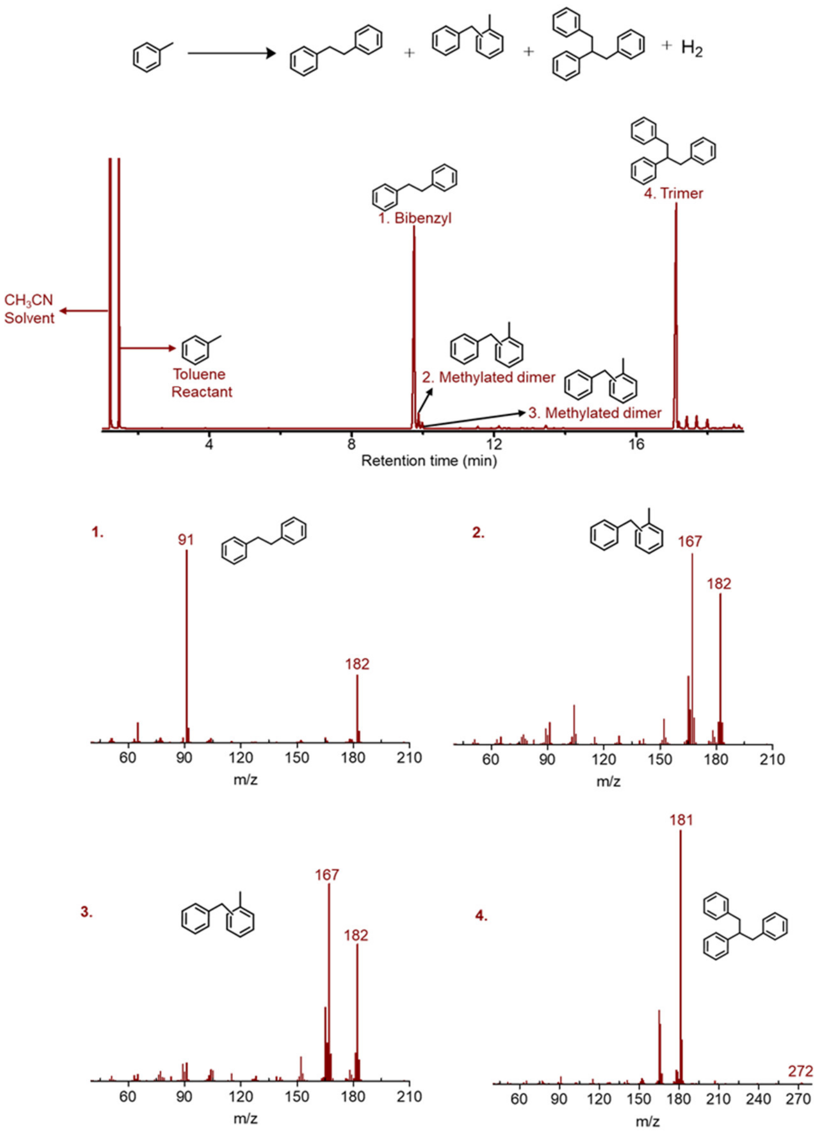

Supplementary Fig. 2 | GC spectrum of product mixtures obtained from the photocatalytic conversion of toluene by $ZnIn_2S_4$ and the mass spectra of major coupling products.

Supplementary Table 2 | Photocatalytic conversion of toluene and the consumed electrons and holes over $ZnIn_2S_4$ and P25.

Catalyst	Coupling products* ($mmol_{benzene\ ring}$)	H_2	Consumed h^+ (mmol)	Consumed e^- (mmol)	e^-/h^+
$ZnIn_2S_4$	0.21	0.10	0.21	0.20	0.95
P25	0.012	0.005	0.012	0.010	0.83

*The unit of $mmol_{benzene\ ring}$ was adopted for coupling products, *i.e.*, bibenzyl, methylated dimers, and trimers. Note that 1 mole of bibenzyl or methylated dimers contain 2 moles of benzene rings, and 1 mol trimers contain 3 moles of benzene rings, thus generation of 1 mole bibenzyl = 2 $mmol_{benzene\ ring}$ coupling products = 2 mmol consumed h^+, generation of 1 mole trimer = 3 $mmol_{benzene\ ring}$ coupling products = 3 mmol consumed h^+.

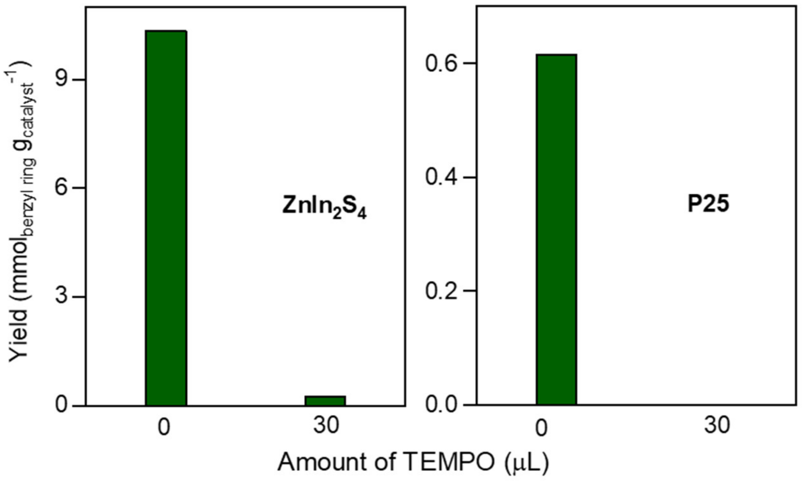

Supplementary Fig. 3 | Photocatalytic conversion of toluene over $ZnIn_2S_4$ (left) and P25 (right) in the absence and presence of TEMPO.

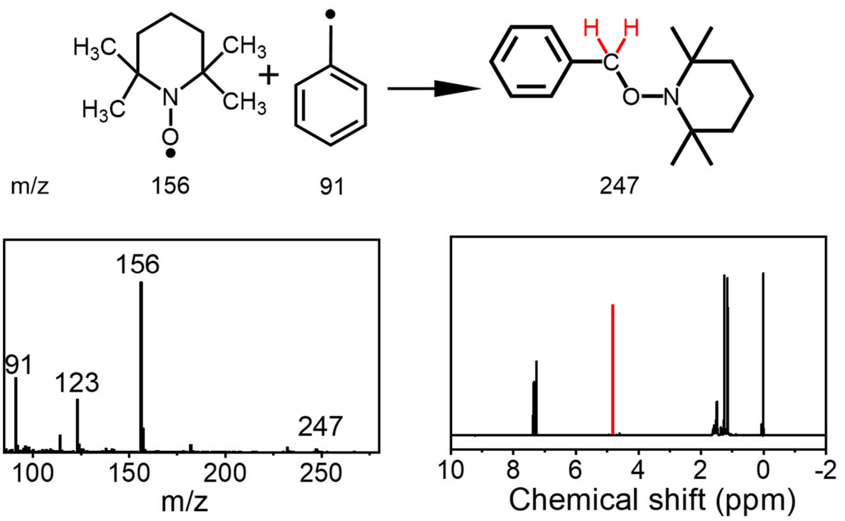

Supplementary Fig. 4 | Mass spectrum (left) and NMR spectrum (right) of product mixture obtained from photocatalytic conversion of toluene in the presence of TEMPO.

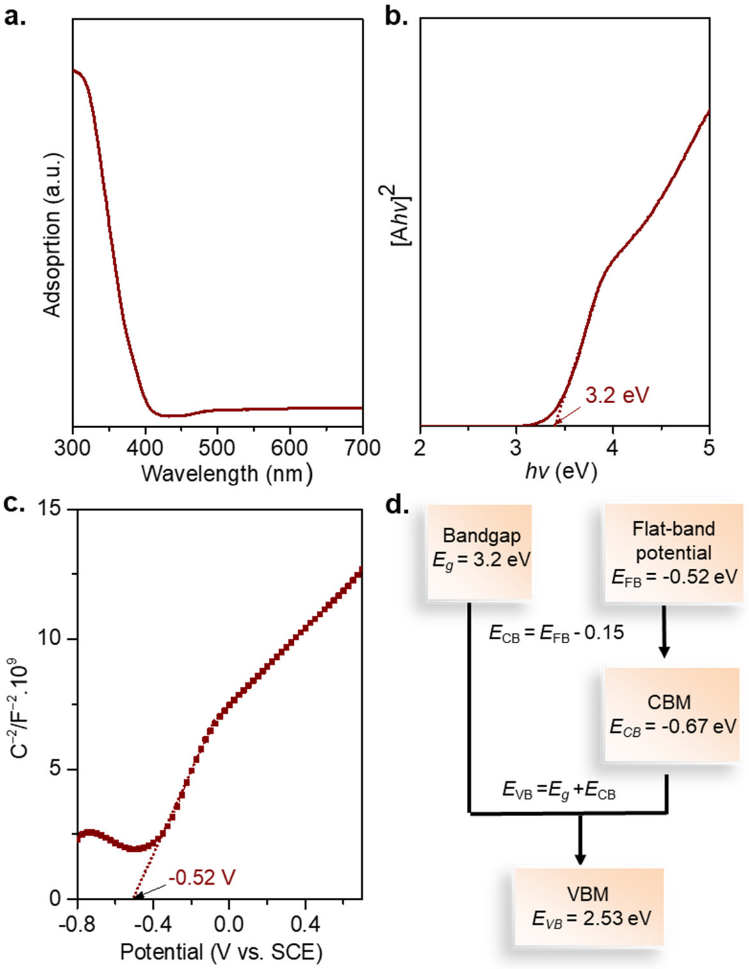

Supplementary Fig. 5 | Determination of P25 band structure. **a,** Diffuse-reflectance UV-vis spectrum. **b,** Plots of modified Kubelka-Munk function *versus* the excitation-light energy. **c,** Mott-Schottky plot. **d,** Schematic illustration for the calculation of valance band position.

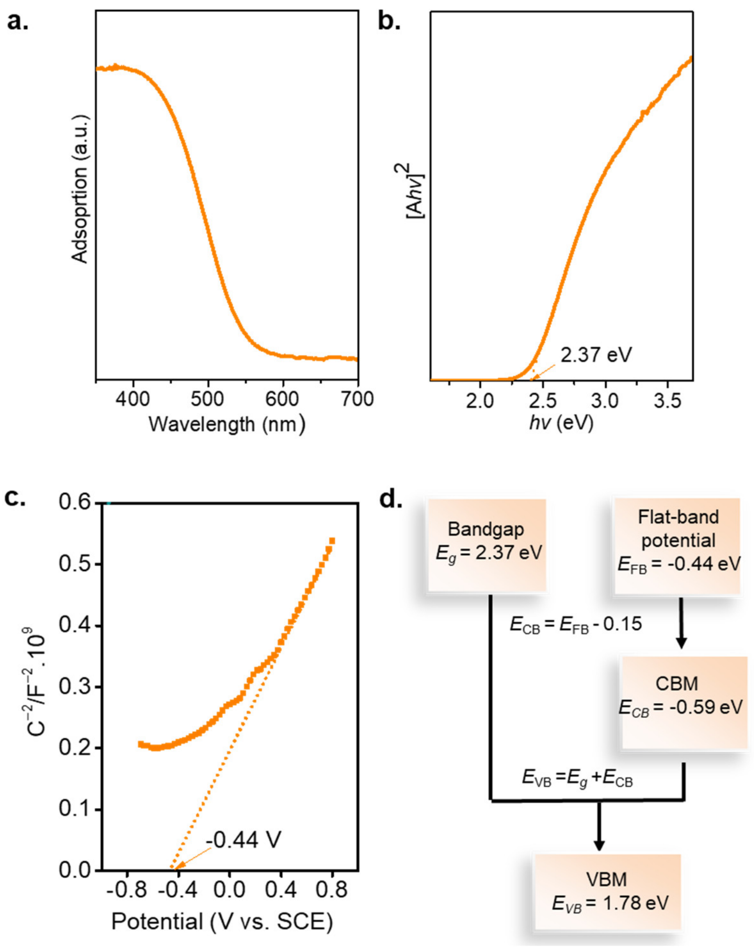

Supplementary Fig. 6 | Determination of $ZnIn_2S_4$ band structure. **a,** Diffuse-reflectance UV-vis spectrum. **b,** Plots of modified Kubelka-Munk function *versus* the excitation light energy. **c,** Mott-Schottky plot. **d,** Schematic illustration for the calculation of valance band position.

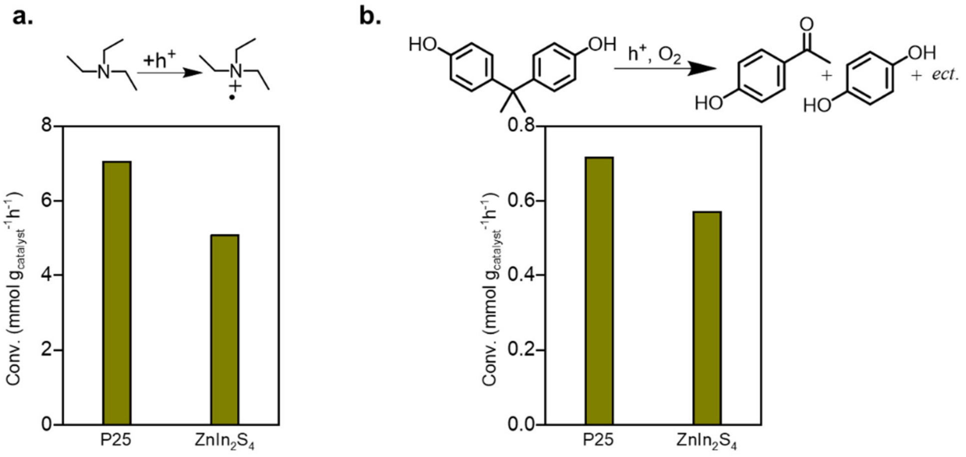

Supplementary Fig. 7 | Performance of P25 and $ZnIn_2S_4$ in electron-transfer-determined reactions. **a.** Photocatalytic conversion of triethylamine. **b.** Photocatalytic decomposition of bisphenol A.

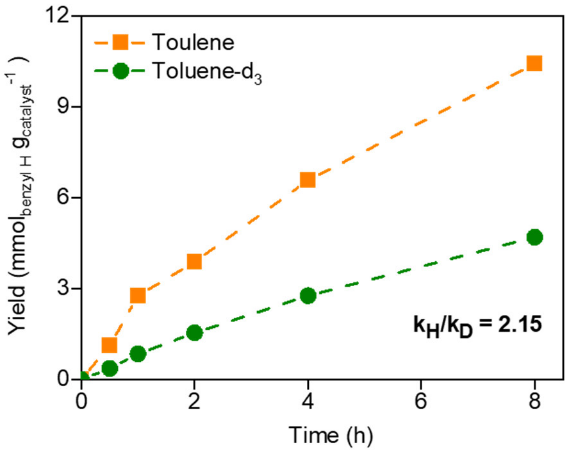

Supplementary Fig. 8 | Apparent kinetic isotopic studies for photocatalytic conversion of toluene and toluene-d_3 over $ZnIn_2S_4$.

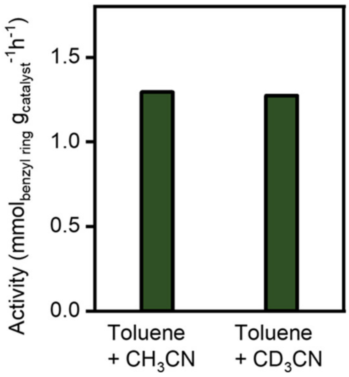

Supplementary Fig. 9 | Comparison of toluene coupling activies over $ZnIn_2S_4$ in CH_3CN and CD_3CN.

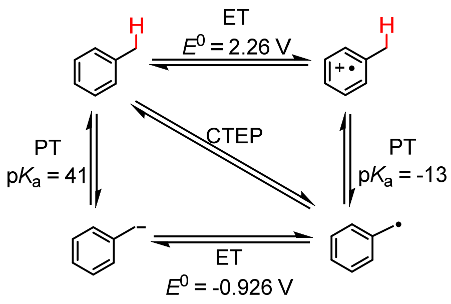

Supplementary Fig. 10 | Square scheme illustrates the thermodynamics for the C−H activation of toluene. The thermodynamic values are from Supplementary Ref. 6.

Supplementary Table 3 | Calculated adsorption energy of $PhCH_3$ and $PhCH_2$• on $ZnIn_2S_4$, $Zn_2In_2S_5$ and TiO_2 in vacuum.

	Adsorption energy (eV)	
	$PhCH_3$	$PhCH_2$•
$ZnIn_2S_4$	-0.61	-1.23
$Zn_2In_2S_5$	-0.59	-1.17
TiO_2	-0.67	-0.74

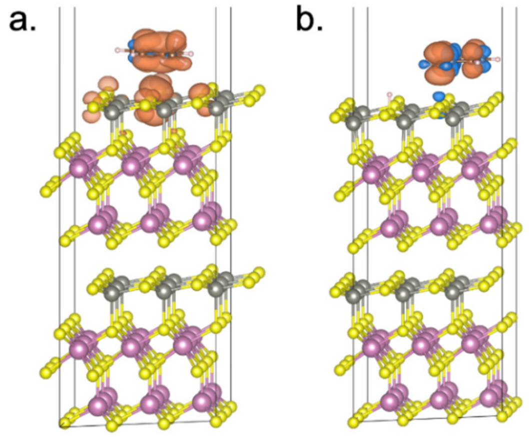

Supplementary Fig. 11 | Spin density of **a,** reactant ($PhCH_3$) state and **b,** product ($PhCH_2$•) state for CPET reaction over the $ZnIn_2S_4$ (001) surface calculated with PBE functional. The grey, yellow, pink, brown, and white balls represent Zn, S, In, C, and H atoms, respectively.

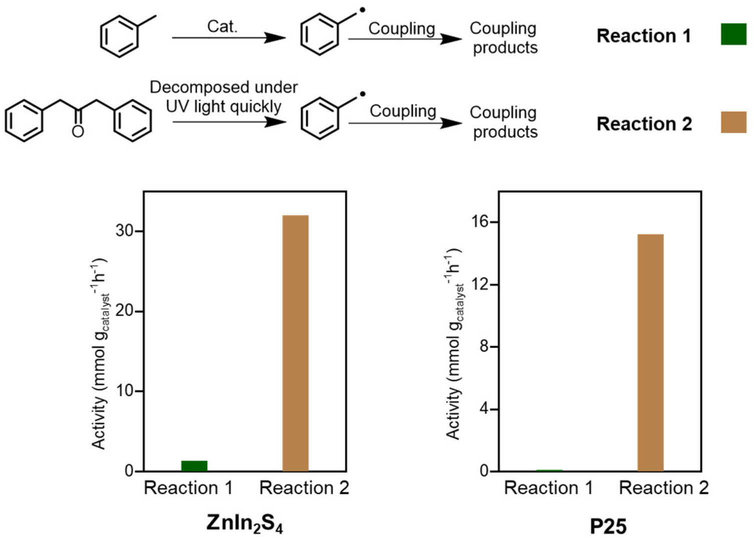

Supplementary Fig. 12 | Control experiments using toluene and dibenzyl ketone as substrates to investigate C−C coupling rates over $ZnIn_2S_4$ and P25.

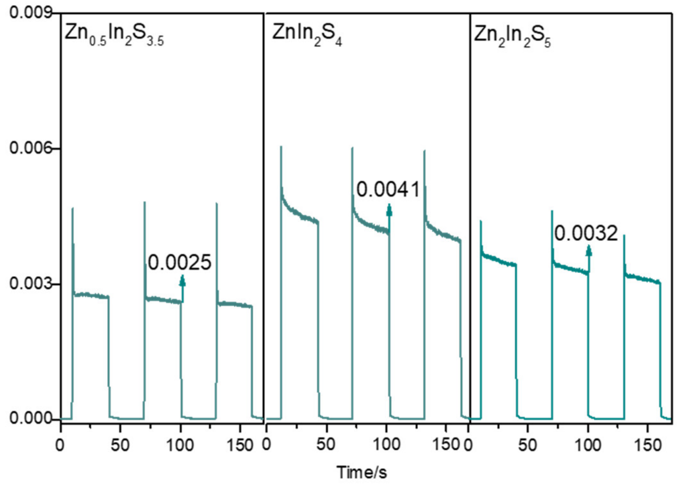

Supplementary Fig. 13 | Transient photocurrent reponses of $Zn_{0.5}In_2S_{3.5}$, $ZnIn_2S_4$, and $Zn_2In_2S_5$.

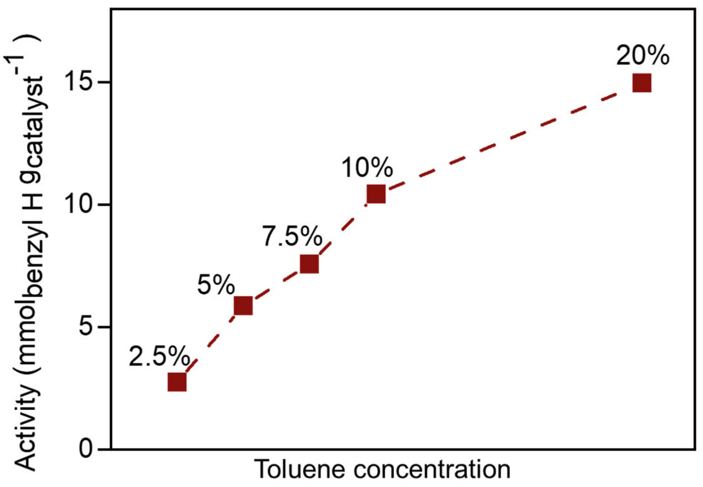

Supplementary Fig. 14 | Change of coupling activity over $Zn_2In_2S_5$ with increasing toluene concentration.

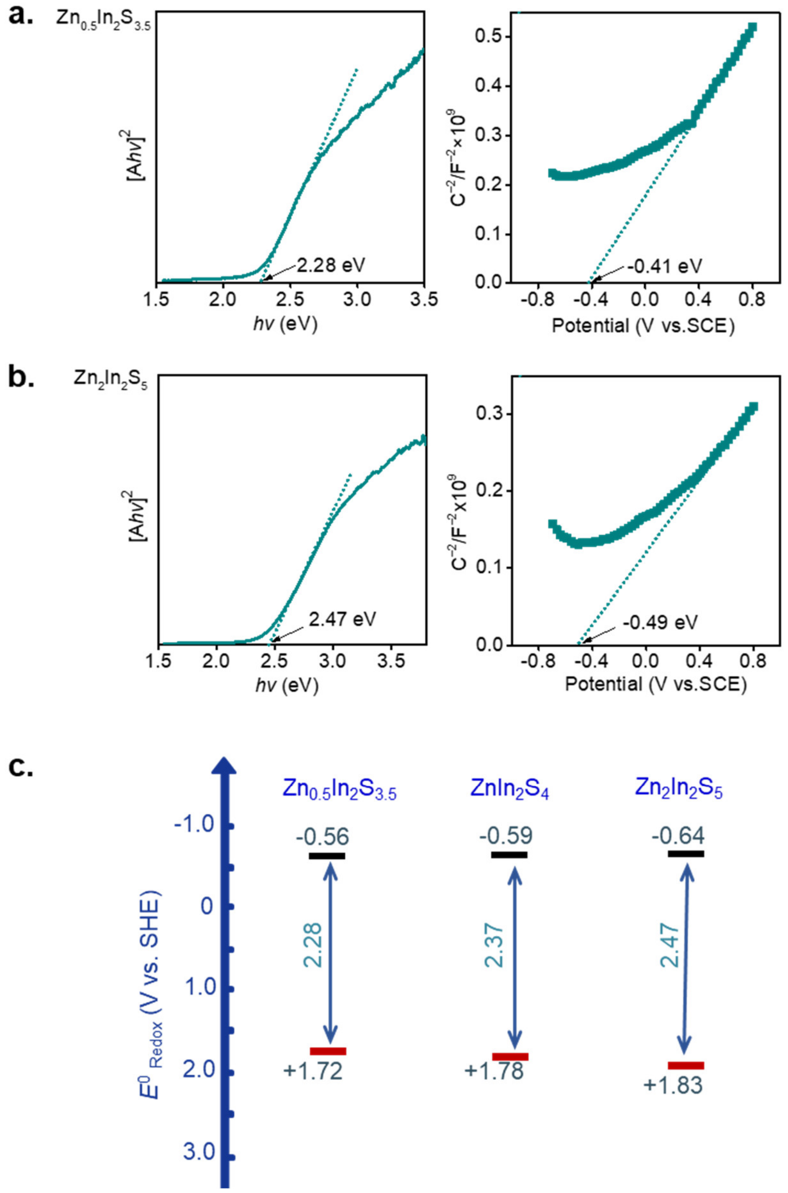

Supplementary Fig. 15 | Plots of modified Kubelka-Munk function *versus* the excitation-light energy and Mott-Schottky plots of **a,** $Zn_{0.5}In_2S_{3.5}$ and **b,** $Zn_2In_2S_5$ as well as **c,** the deduced band edge positions of $Zn_{0.5}In_2S_{3.5}$, $ZnIn_2S_4$, and $Zn_2In_2S_5$.

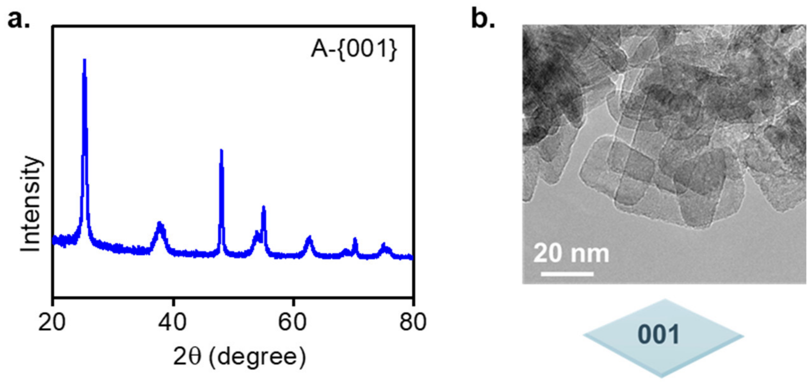

Supplementary Fig. 16 | a, XRD patterns and **b,** TEM micrographs and schematic morphology of A-{001}.

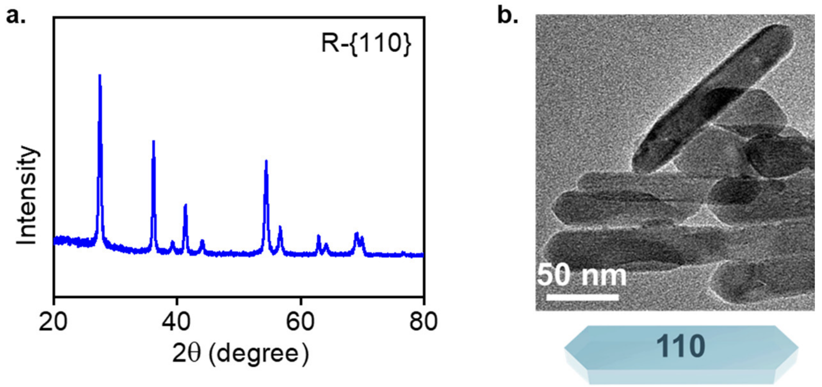

Supplementary Fig. 17 | a, XRD patterns and **b,** TEM micrographs and schematic morphology of R-{110}.

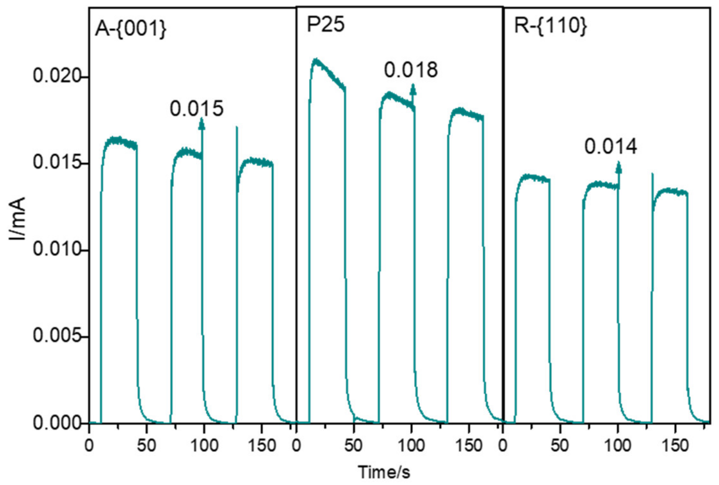

Supplementary Fig. 18 | Transient photocurrent responses of A-{001}, P25, and R-{110}.

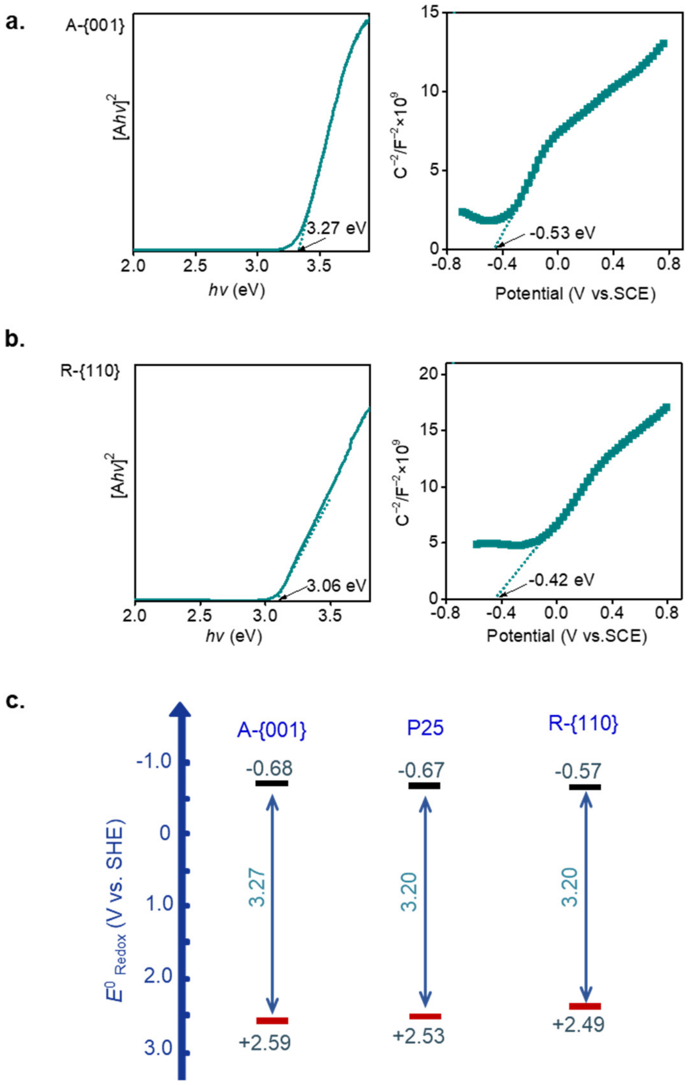

Supplementary Fig. 19 | Plots of modified Kubelka-Munk function *versus* the excitation-light energy and Mott-Schottky plots of **a,** A-{001} and **b,** R-{110} as well as **c,** the deduced band edge positions of A-{001}, P25, and R-{110}.

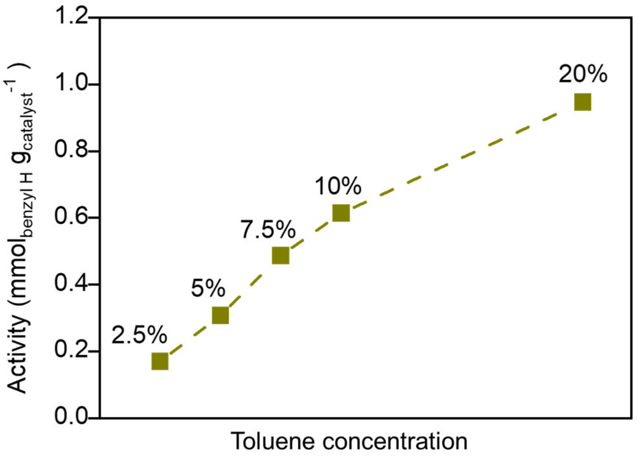

Supplementary Fig. 20 | Change of coupling activity over P25 with increasing toluene concentration.

Supplementary Table 4 | Photocatalytic coupling of fossil- and biomass-derived chemicals by $Zn_2In_2S_5$.

No.	Reactant	Conv. (%)	Yield (%)	
			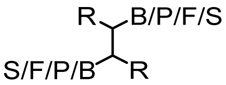<br>*	Methylated products+ Trimers+ Others
1	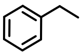	73	65	7
2	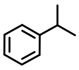	85	79	4
3	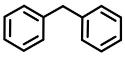	72	69	3
4	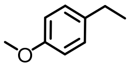 	80	76	3
5	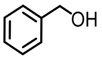 	99	91	3
6	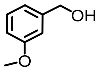 	97	82	2
7	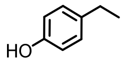 	97	61	17
8	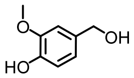 	95	86	0
9	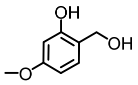 	96	79	0
10	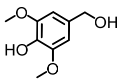 	79	74	0
11	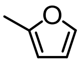 	73	23	48
12	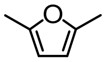 	97	30	50
13	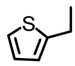 	96	66	25
14	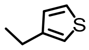 	98	73	19
15	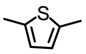 	81	34	37
16	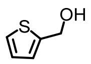 	96	53	7

*See Supplementary Fig. 21-26, 29-32, and 34-39 for structures and mass spectra of the products.

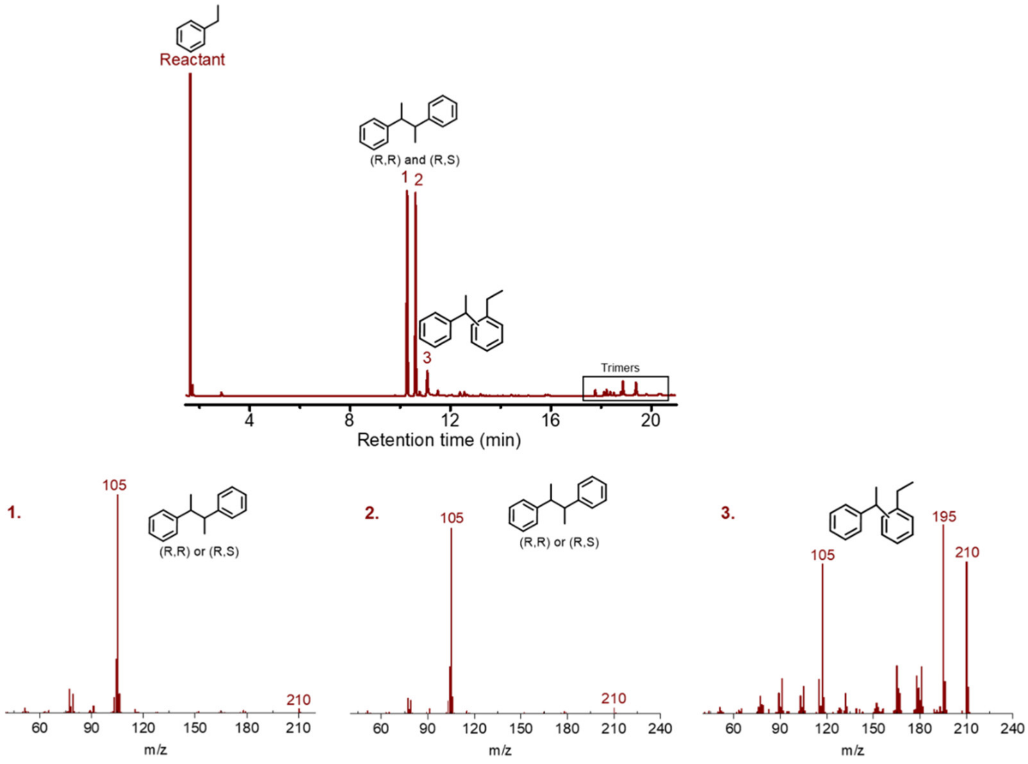

Supplementary Fig. 21 | GC spectrum of product mixtures obtained from the photocatalytic conversion of ethylbenzene and the mass spectra of major coupling products.

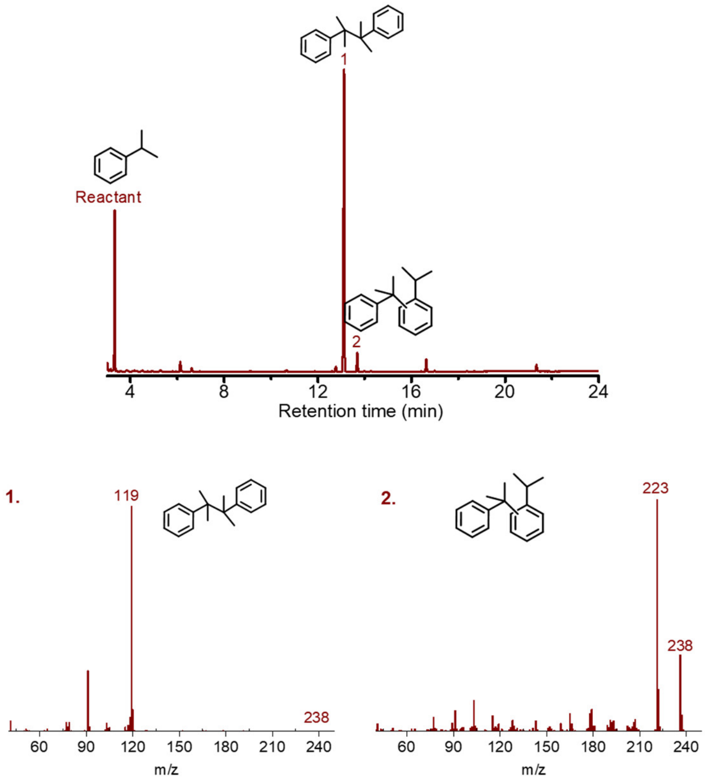

Supplementary Fig. 22 | GC spectrum of product mixtures obtained from the photocatalytic conversion of cumene and the mass spectra of major coupling products.

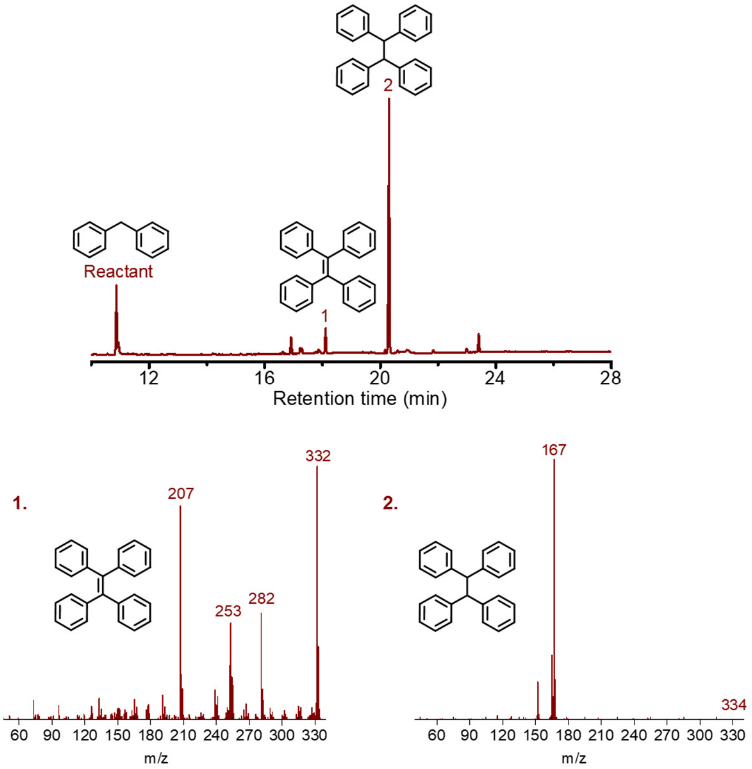

Supplementary Fig. 23 | GC spectrum of product mixtures obtained from the photocatalytic conversion of diphenylmethane and the mass spectra of major coupling products.

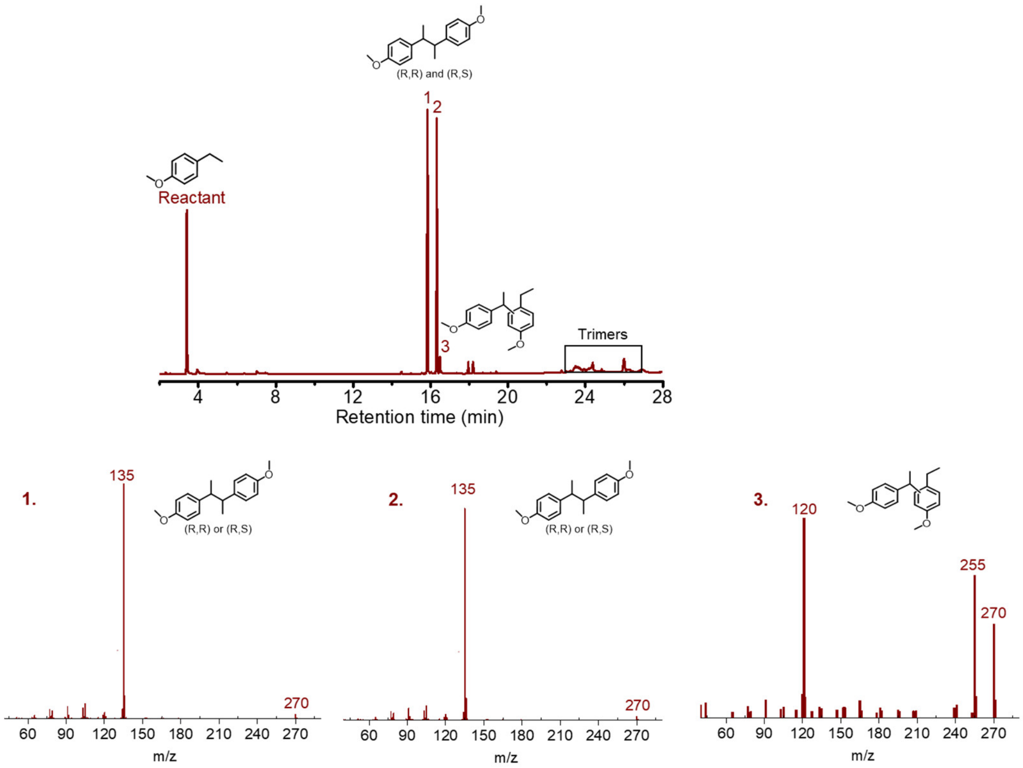

Supplementary Fig. 24 | GC spectrum of product mixtures obtained from the photocatalytic conversion of 4-ethylanisole and the mass spectra of major coupling products.

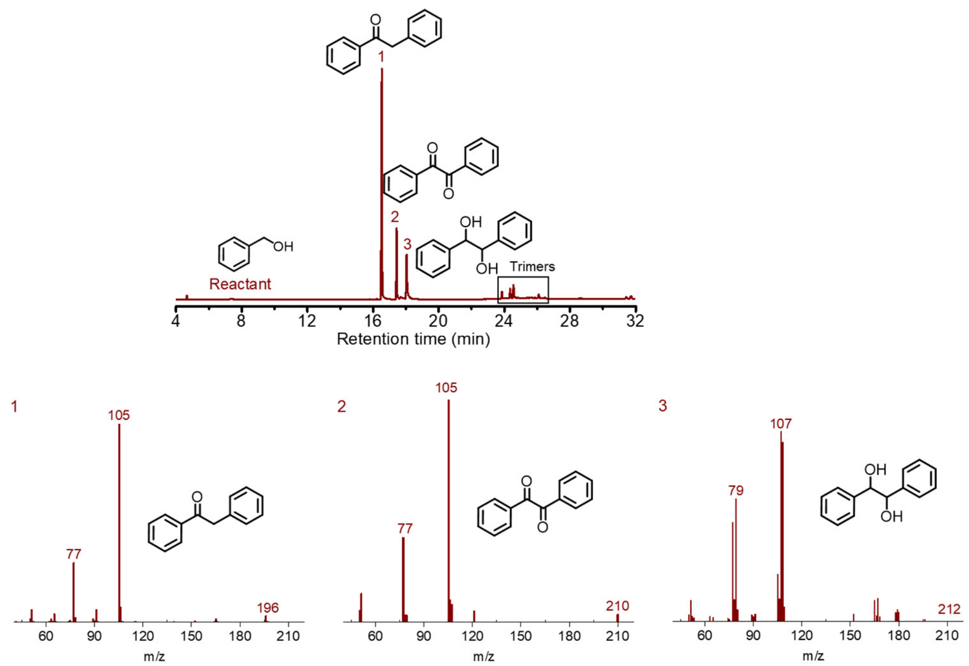

Supplementary Fig. 25 | GC spectrum of product mixtures obtained from the photocatalytic conversion of benzyl alcohol and the mass spectra of major coupling products. Products 1-3 are grouped into A, while trimers are grouped into B in Fig. 5 main text.

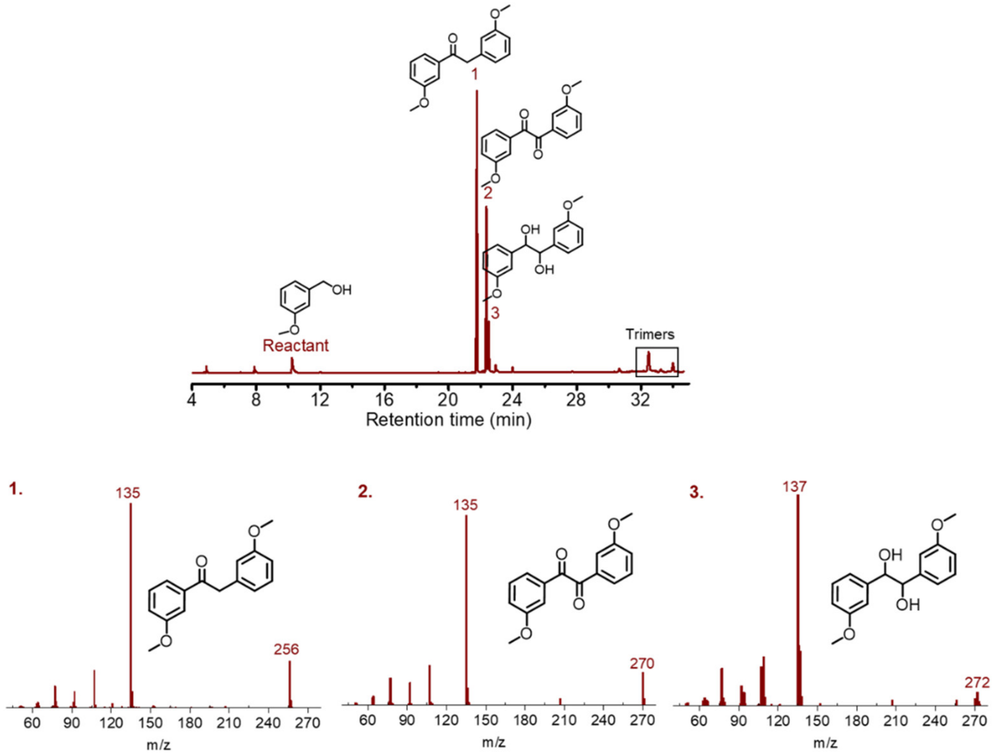

Supplementary Fig. 26 | GC spectrum of product mixtures obtained from the photocatalytic conversion of 3-methoxybenzyl alcohol and the mass spectra of major coupling products. Products 1-3 are grouped into A, while trimers are grouped into B in Fig. 5 main text.

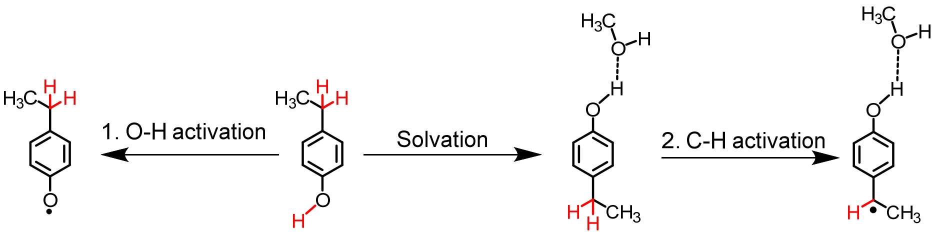

Supplementary Fig. 27 | Proposed mechanism for the role of protonic solvent (using CH_3OH as an example) in the selective activation of the C−H bond in phenolic chemicals.

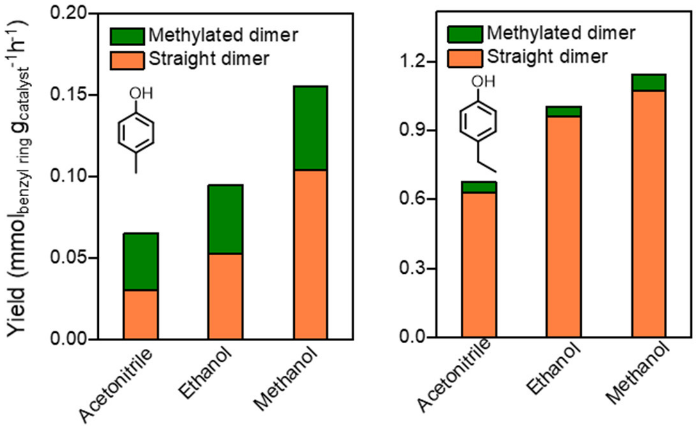

Supplementary Fig. 28 | Photocatalytic conversion of phenolic chemicals, *i.e.*, *p*-cresol and 4-ethyl-phenol, in acetonitrile, ethanol, and methanol.

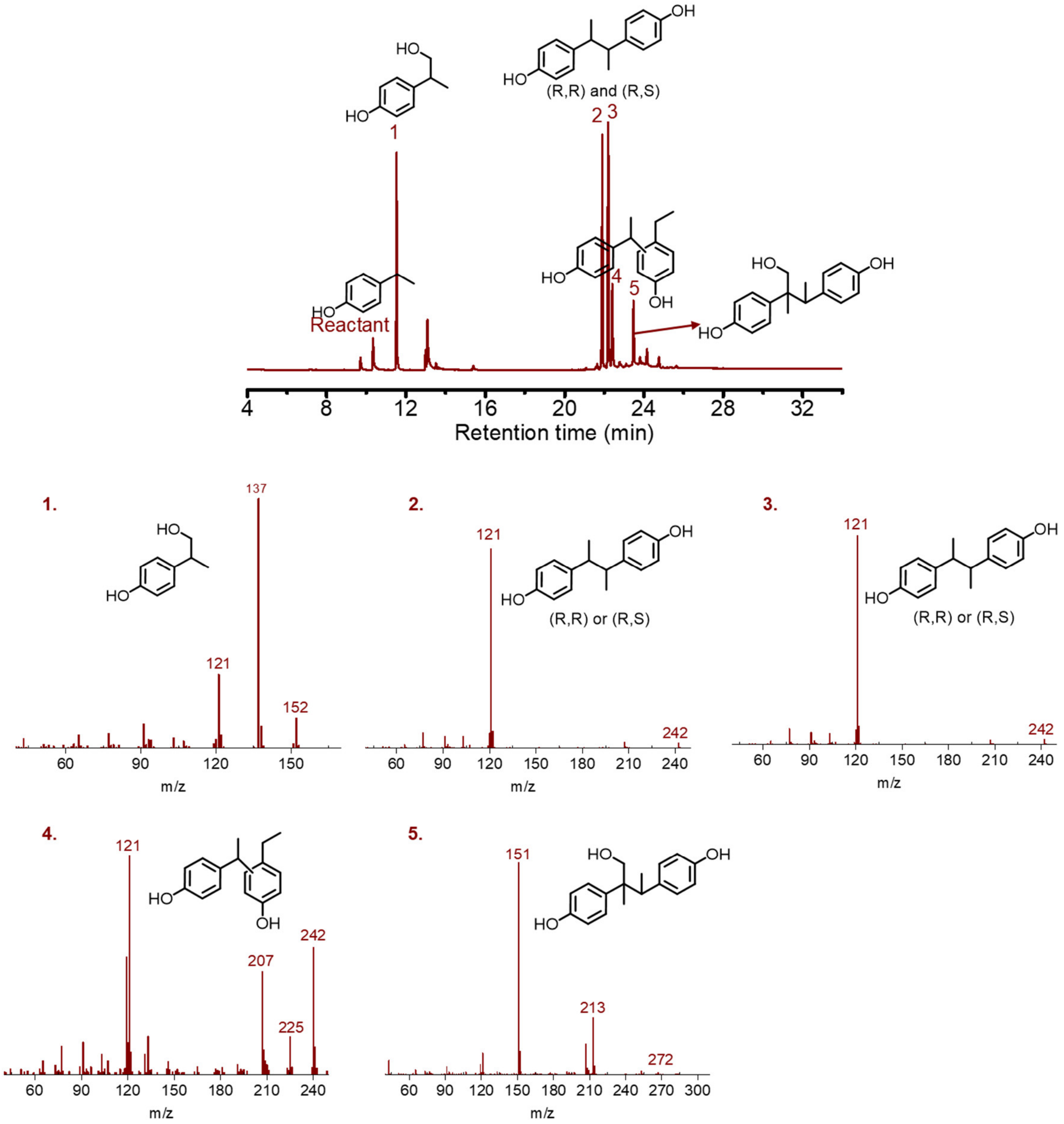

Supplementary Fig. 29 | GC spectrum of product mixtures obtained from the photocatalytic conversion of 4-ethyl phenol and the mass spectra of major coupling products. Chemical 1 is the coupling product from 4-ethylphenol and the solvent methanol, and chemicals 2-5 are self-coupling products from 4-ethylphenol and chemical 1. Products 2, 3, and 5 are grouped into A, while products 1 and 4 are grouped into B in Fig. 5 main text.

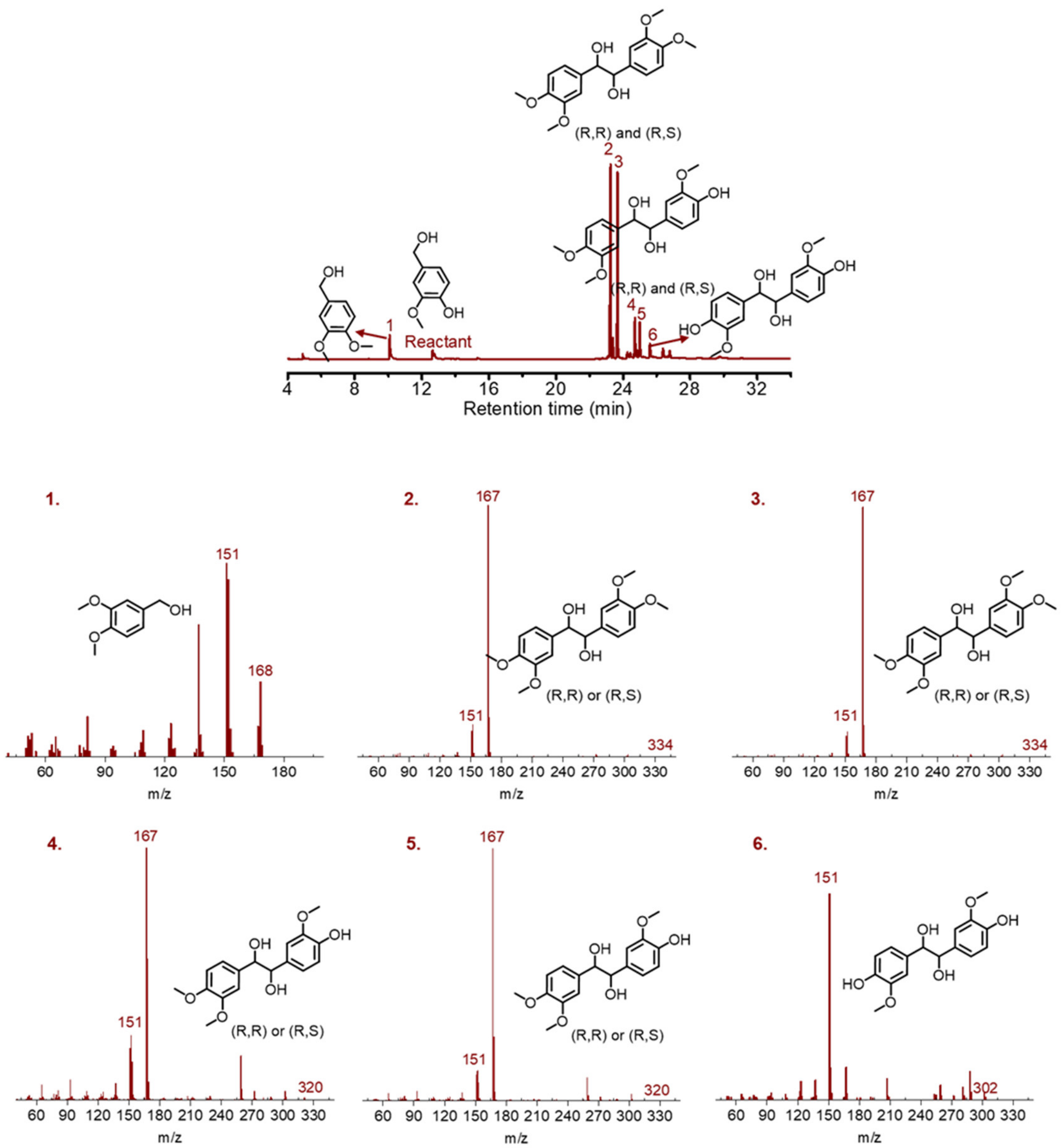

Supplementary Fig. 30 | GC spectrum of product mixtures obtained from the photocatalytic conversion of 4-hydroxy-3-methoxybenzyl alcohol and the mass spectra of major products. Chemical 1 is generated from etherification between 4-hydroxy-3-methoxybenzyl alcohol and the solvent methanol and thus is not a coupling product. Products 2-6 are grouped into A in Fig. 5 main text.

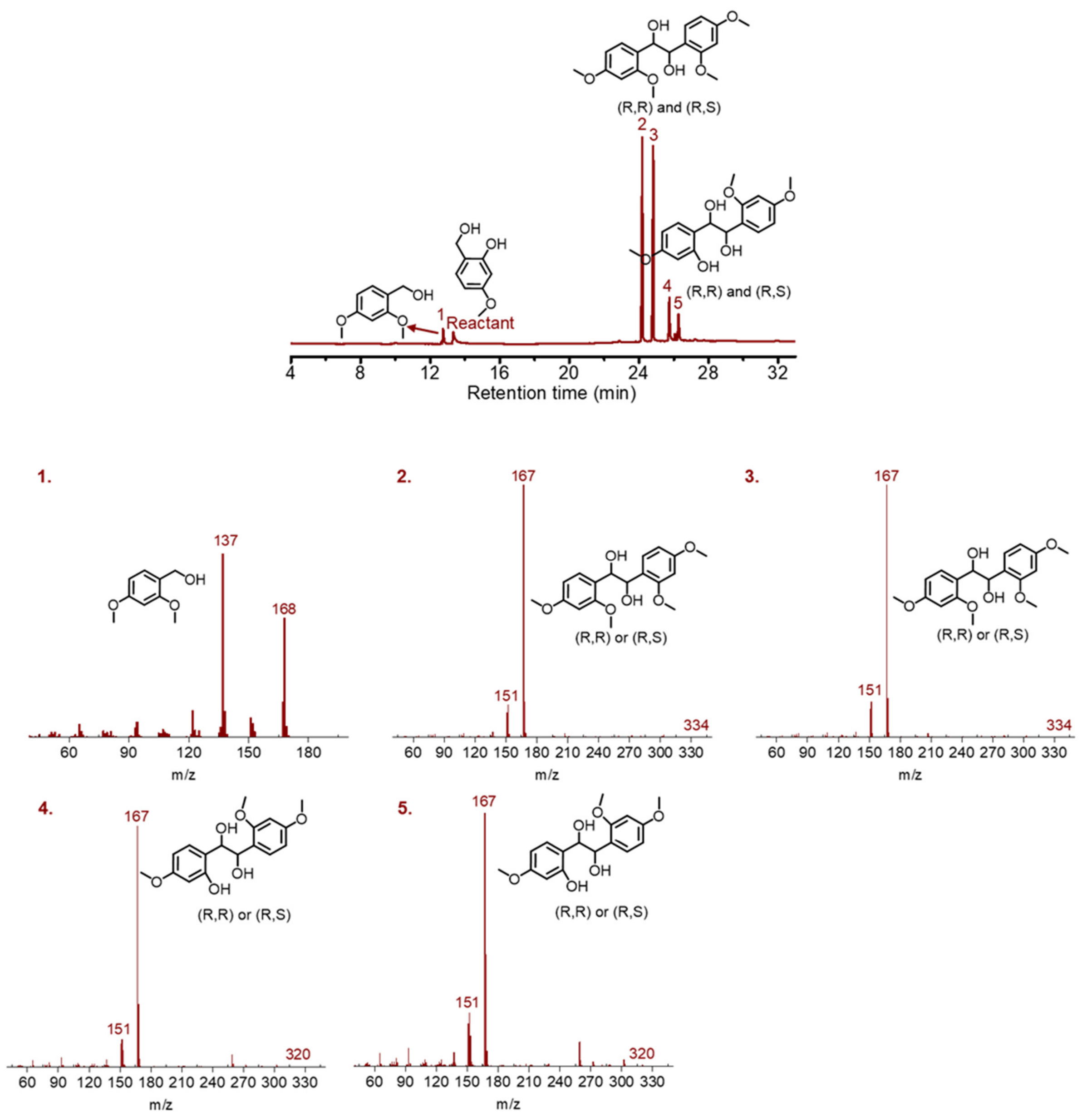

Supplementary Fig. 31 | GC spectrum of product mixtures obtained from the photocatalytic conversion of 2-hydroxy-4-methoxybenzyl alcohol and the mass spectra of major products. Chemical 1 is generated from etherification between 4-hydroxy-3-methoxybenzyl alcohol and the solvent methanol and thus is not considered a coupling product. Products 2-5 are grouped into A in Fig. 5 main text.

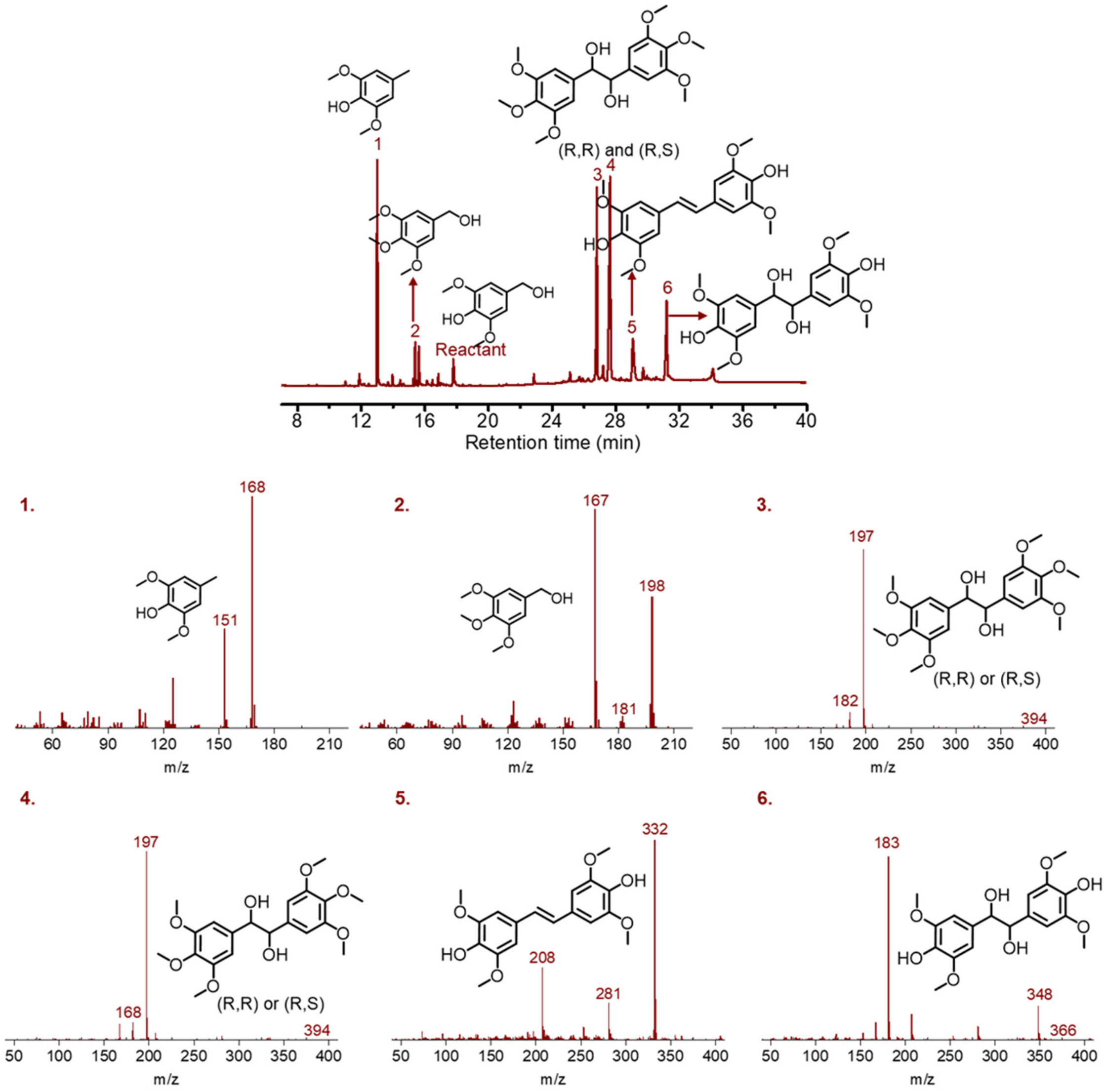

Supplementary Fig. 32 | GC spectrum of product mixtures obtained from the photocatalytic conversion of 4-hydroxy-3,5-dimethoxybenzyl alcohol and the mass spectra of major products. Chemicals 1 and 2 are not coupling products. Chemicals 3-6 are coupling products and are grouped into A in Fig. 5 main text.

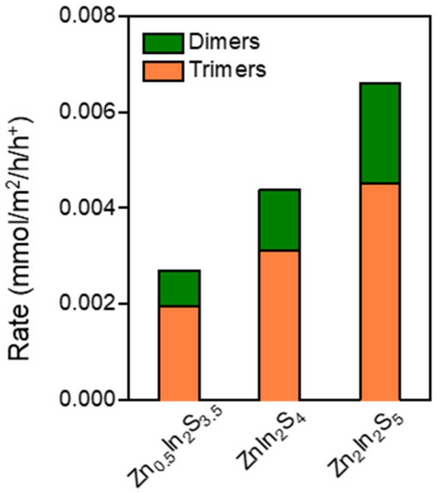

Supplementary Fig. 33 | Conversion of 2-methylfuran over $Zn_{0.5}In_2S_{3.5}$, $ZnIn_2S_4$, and $Zn_2In_2S_5$. The reaction rate is expressed in mmol/m²/h/h_s^+ to account for variations in the intrinsic electron efficiency of the catalysts, which is evaluated by photocurrent measurements from Fig. S11, and the intensity of $Zn_{0.5}In_2S_{3.5}$ is normalized as 1 unit of h_s^+.

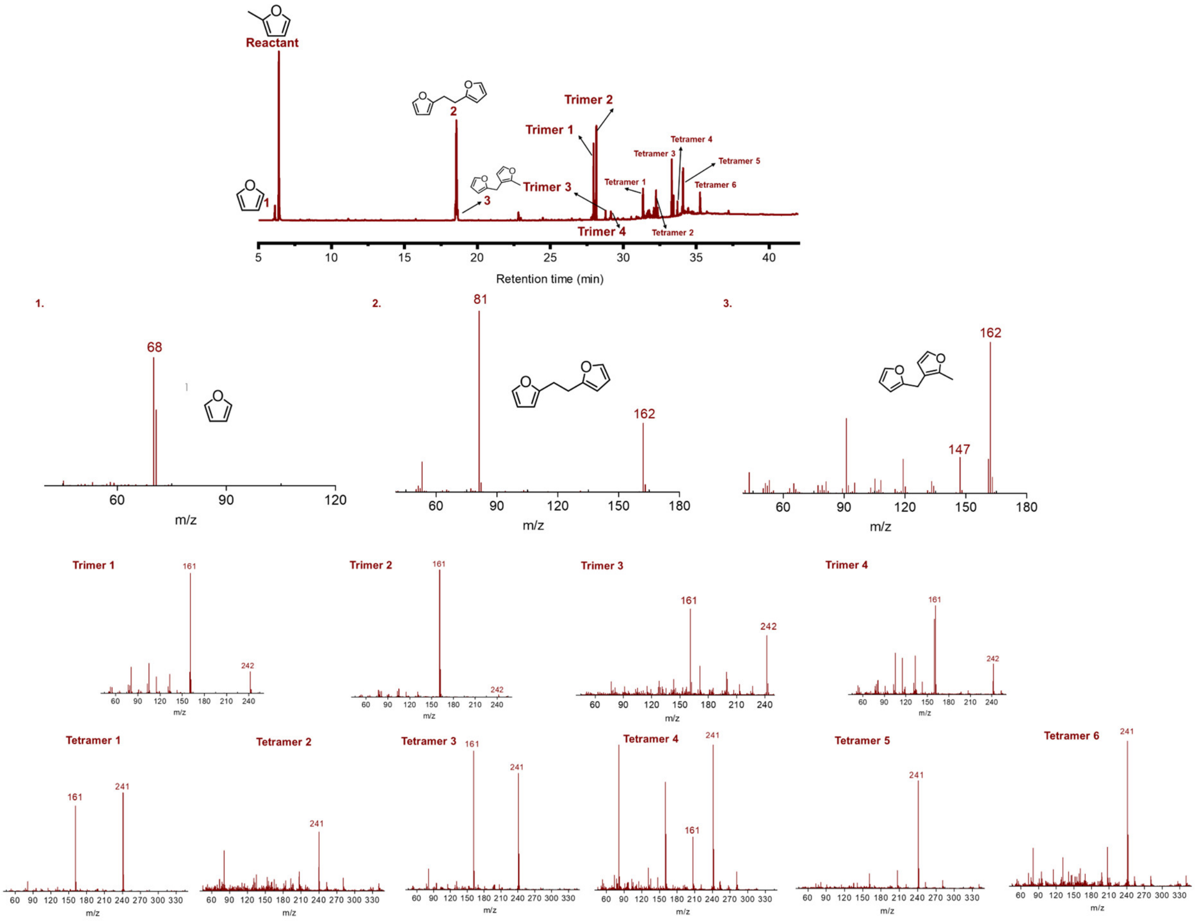

Supplementary Fig. 34 | GC spectrum of product mixtures obtained from the photocatalytic conversion of 2-methylfuran and the mass spectra of major products. Extending the reaction time for methylfuran to 36 hours led to trimers and tetramers as the predominant products. There are a lot of diffenect coupling possibilities for trimers and tetramers, and these compounds exhibit similar peak characteristics (m/z = 161 for trimers; m/z = 241 for tetramers). Consequently, only representative mass spectra for these major products are showcased.

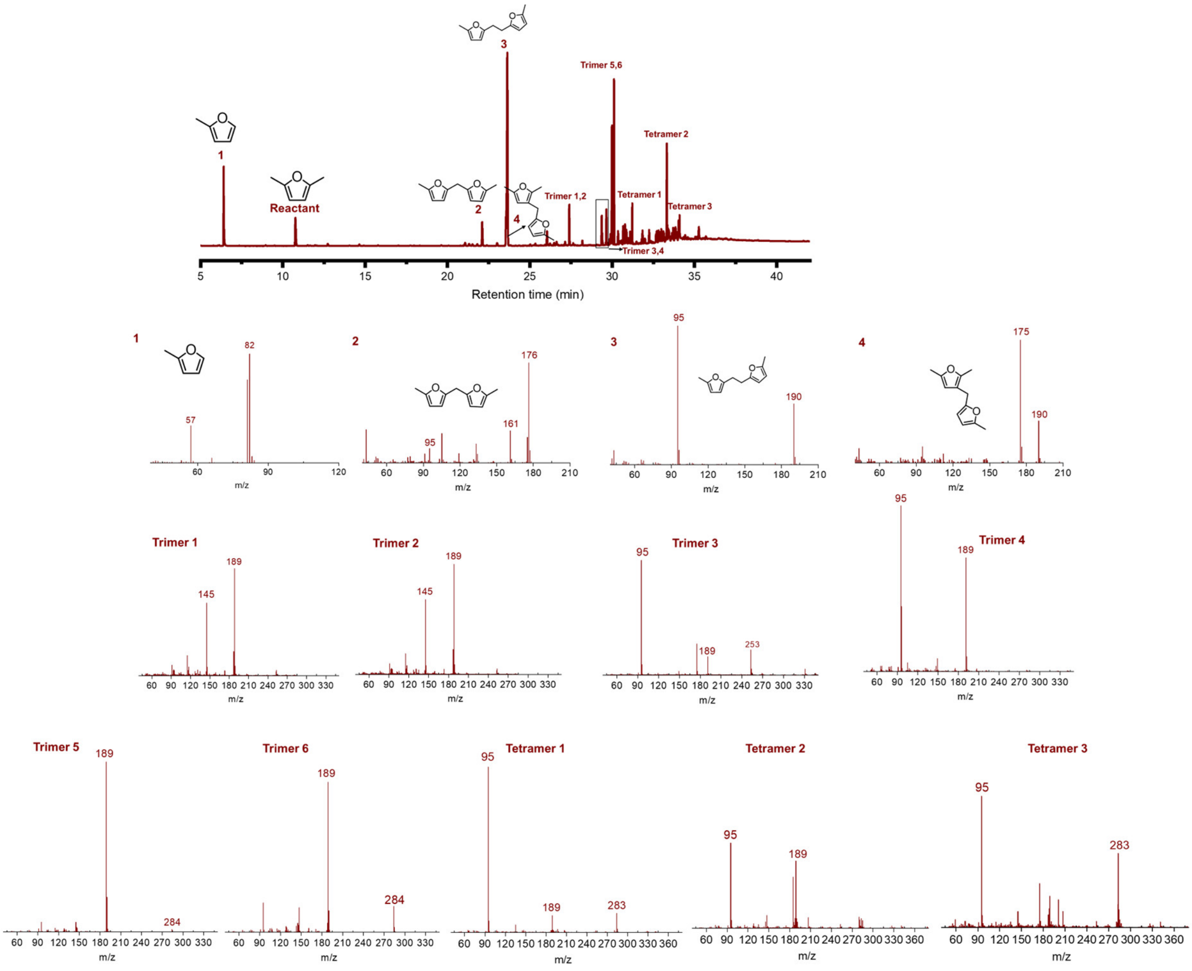

Supplementary Fig. 35 | GC spectrum of product mixtures obtained from the photocatalytic conversion of 2,5-dimethylfuran and the mass spectra of major products. Extending the reaction time for 2,5-dimethylfuran to 36 hours led to trimers and tetramers as the predominant products. There are a lot of diffenect coupling possibilities for trimers and tetramers, and these compounds exhibit similar peak characteristics (m/z = 189 for trimers; m/z = 283 for tetramers). Consequently, only representative mass spectra for these major products are showcased.

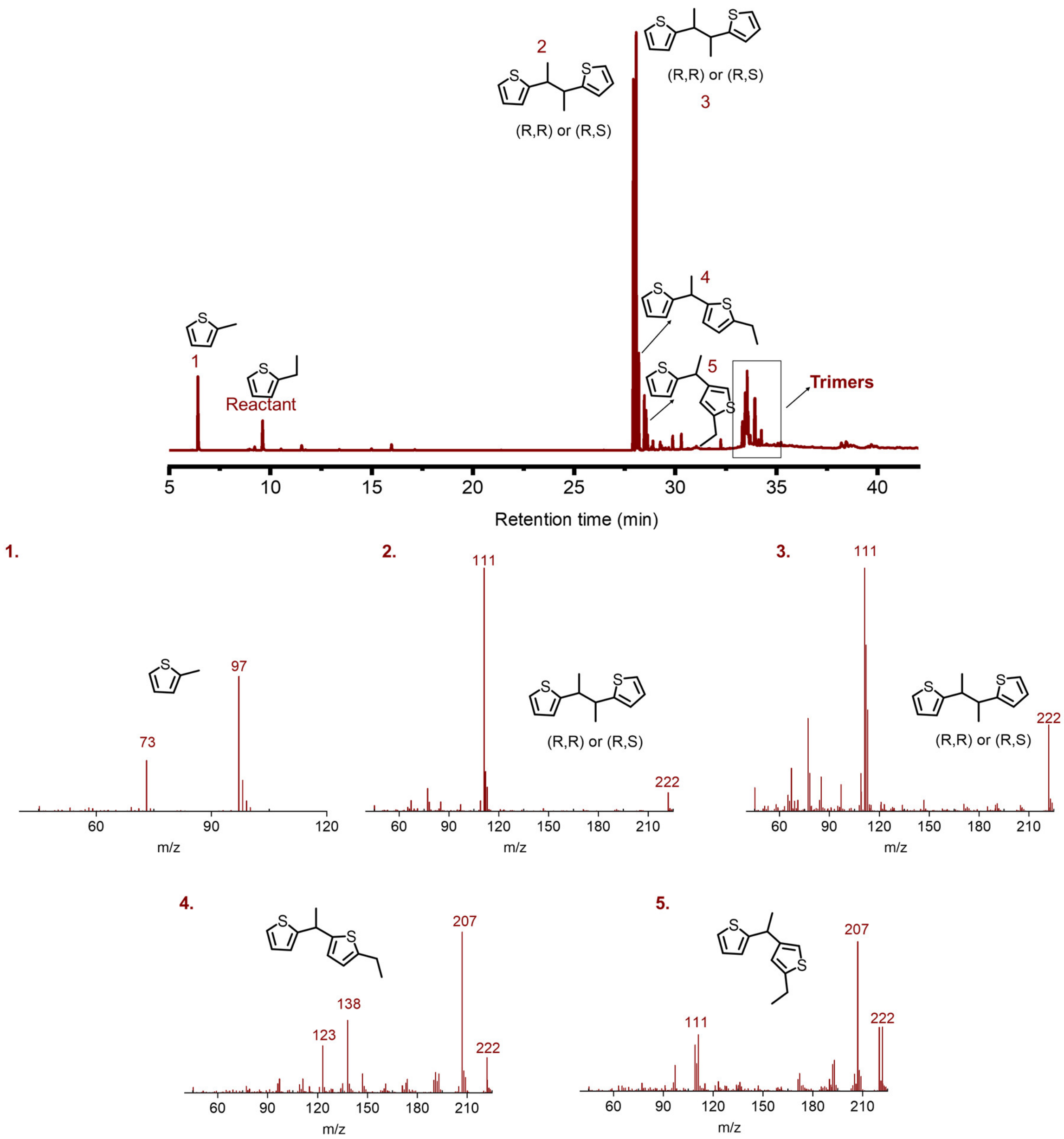

Supplementary Fig. 36 | GC spectrum of product mixtures obtained from the photocatalytic conversion of 2-ethyl thiophene and the mass spectra of major products.

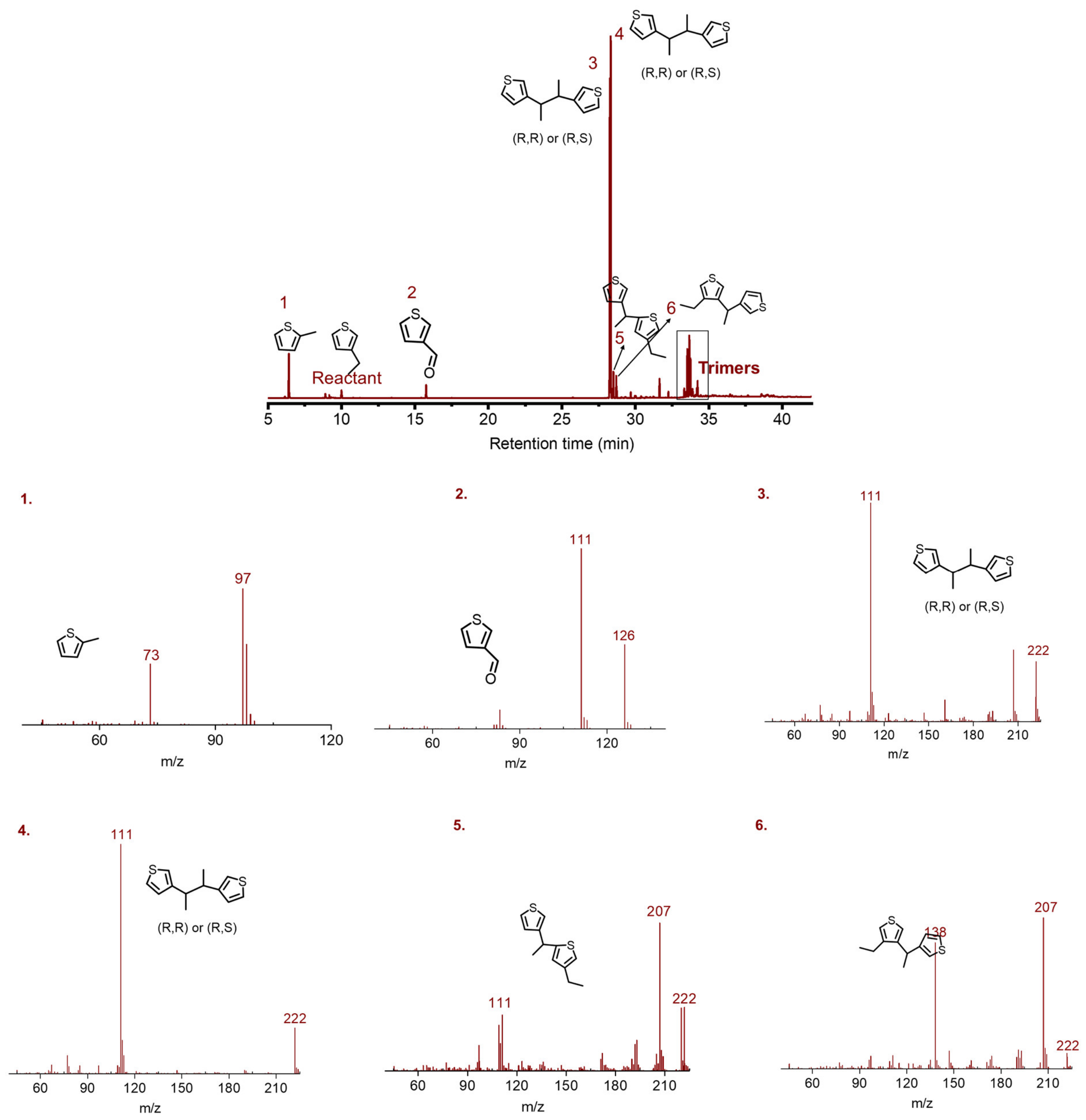

Supplementary Fig. 37 | GC spectrum of product mixtures obtained from the photocatalytic conversion of 3-ethyl thiophene and the mass spectra of major products.

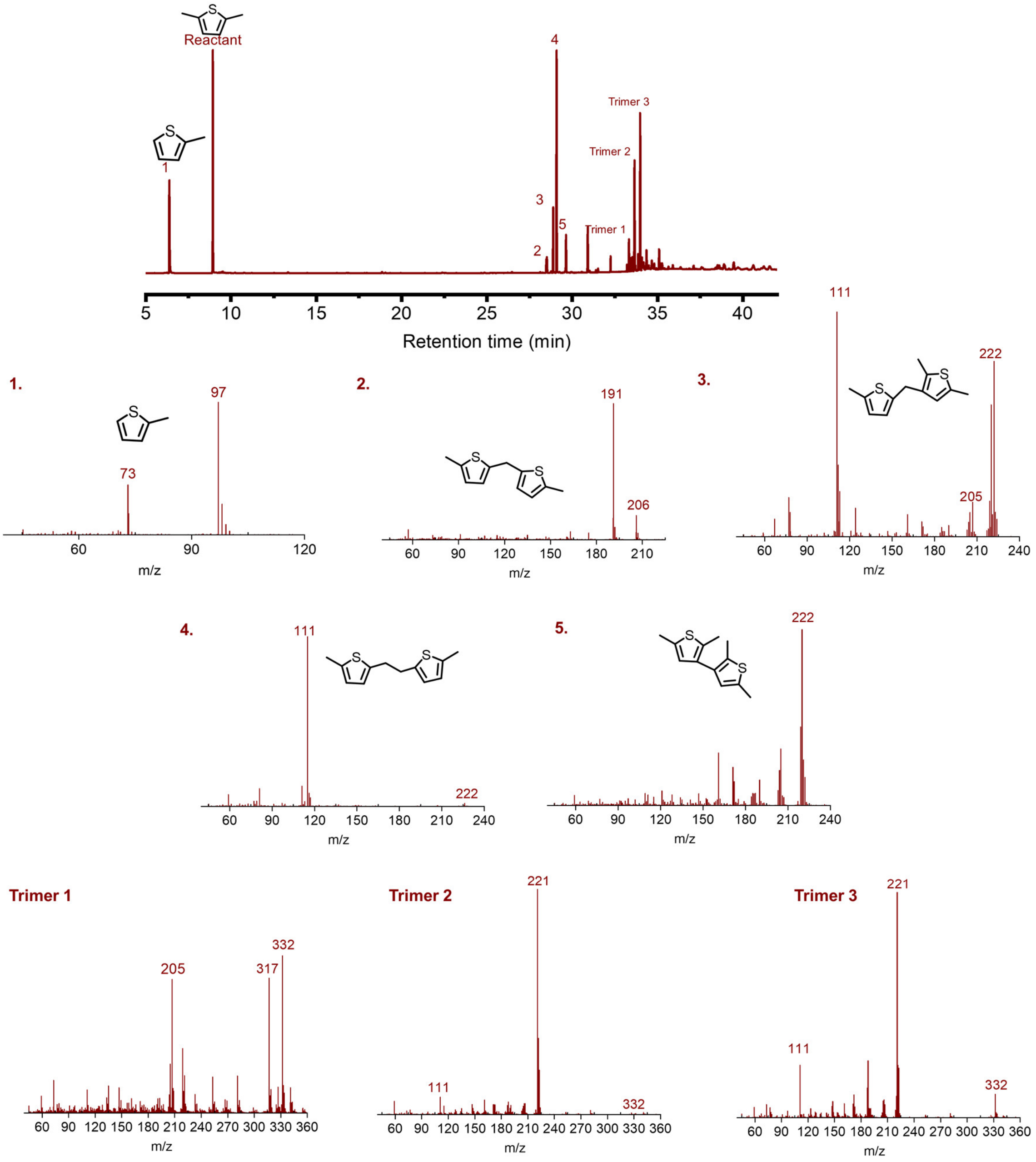

Supplementary Fig. 38 | GC spectrum of product mixtures obtained from the photocatalytic conversion of 2,5-dimethyl-thiophene and the mass spectra of major products.

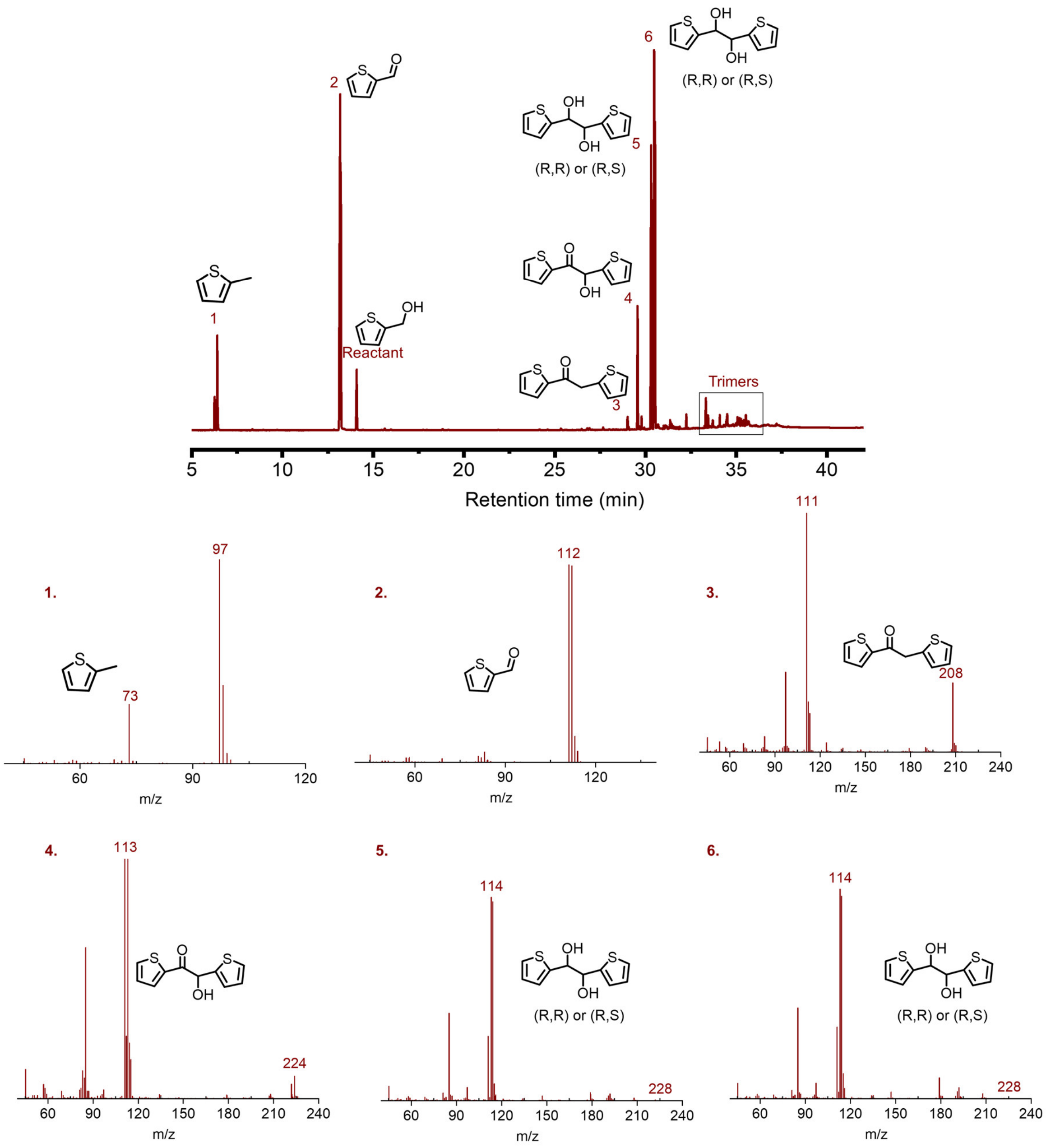

Supplementary Fig. 39 | GC spectrum of product mixtures obtained from the photocatalytic conversion of 2-methanol thiophene and the mass spectra of major products.

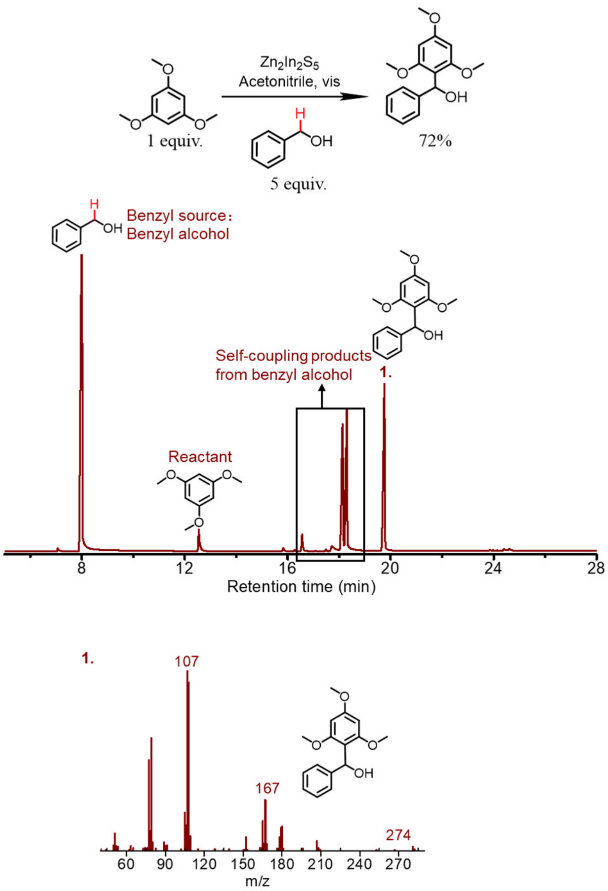

Supplementary Fig. 40 | GC spectrum of product mixtures obtained from the photocatalytic coupling of 1,3,5-trimethoxybenzene with benzyl alcohol and the mass spectrum of the major product based on 1,3,5-trimethoxybenzene.

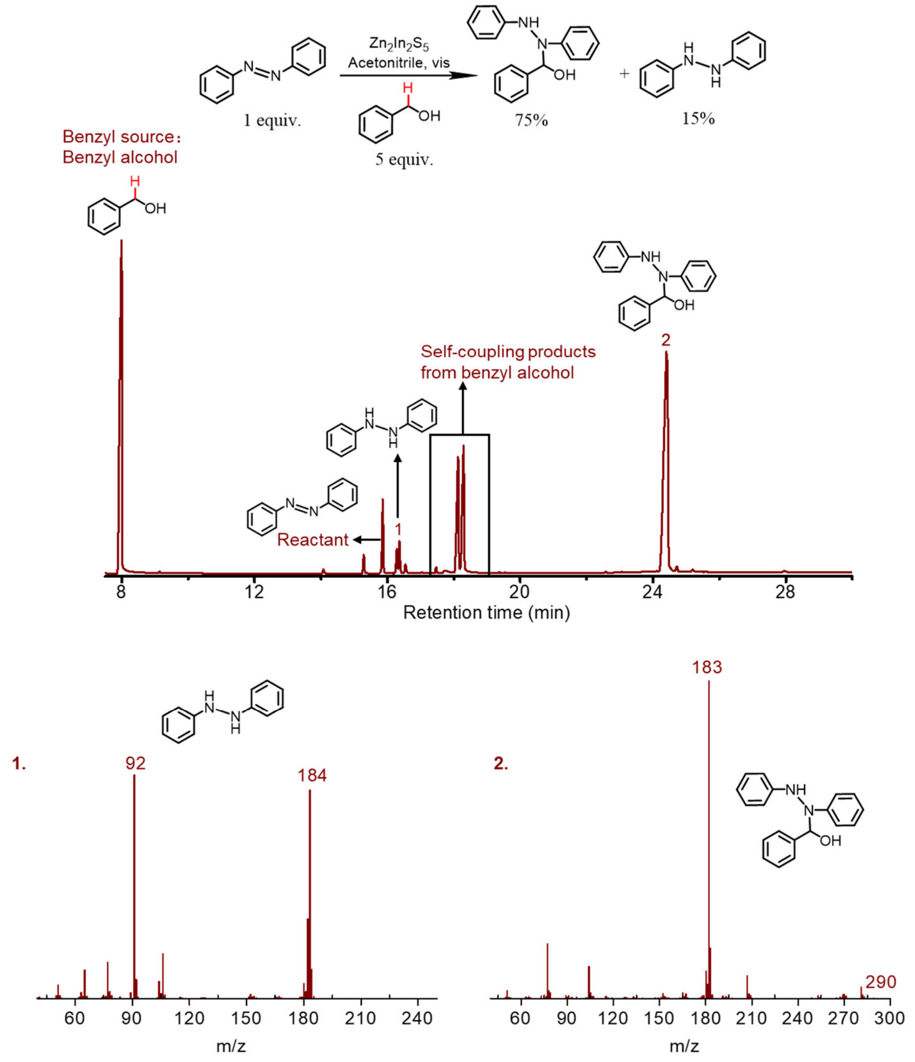

Supplementary Fig. S41 | GC spectrum of product mixtures obtained from the photocatalytic coupling of azobenzene with benzyl alcohol and the mass spectra of major products based on azobenzene.

Supplementary References

1. Kresse, G. & Joubert, D. From ultrasoft pseudopotentials to the projector augmented-wave method. *Phys. Rev. B: Condens. Matter Mater. Phys.* **59**, 1758−1775 (1999).
2. Kresse, G. & Furthmüller, J. Efficiency of ab-initio total energy calculations for metals and semiconductors using a plane-wave basis set. *Comput. Mater. Sci.* **6**, 15−50 (1996).
3. Kresse, G. & Hafner, J. Ab initio molecular dynamics for liquid metals. *Phys. Rev. B: Condens. Matter Mater. Phys.* **47**, 558−561 (1993).
4. Grimmea, S., Antony, J., Ehrlich, S. & Krieg, H. A consistent and accurate ab initio parametrization of density functional dispersion correction (DFT-D) for the 94 elements H-Pu. *J. Chem. Phys.* **132**, 154104 (2010).
5. Perdew, J. P., Burke, K. & Ernzerhof, M. Generalized gradient approximation made simple. *Phys. Rev. Lett.* **77**, 3865−3868 (1996).
6. Mazzarella, D. Crisenza, G. E. M. & Melchiorre, P. Asymmetric photocatalytic C–H functionalization of toluene and derivatives. *J. Am. Chem. Soc.* **140**, 8439-8443 (2018).
