# Supplementary material for: Zinc-indium-sulfide favors efficient C − H bond activation by concerted proton-coupled electron transfer
Source: Nat Commun. 2024 Jun 11;15:4967. doi: 10.1038/s41467-024-49265-2 (PMC11167015; doi:10.1038/s41467-024-49265-2)
Supplement: Supplementary file 3 — Reporting Summary [file 41467_2024_49265_MOESM3_ESM.pdf]

nature portfolio

Corresponding author(s): Bert Sels

Last updated by author(s): May 5, 2024

Reporting Summary

Nature Portfolio wishes to improve the reproducibility of the work that we publish. This form provides structure for consistency and transparency in reporting. For further information on Nature Portfolio policies, see our Editorial Policies and the Editorial Policy Checklist.

Statistics

For all statistical analyses, confirm that the following items are present in the figure legend, table legend, main text, or Methods section.

n/a	Confirmed	
☒	☐	The exact sample size (*n*) for each experimental group/condition, given as a discrete number and unit of measurement
☐	☒	A statement on whether measurements were taken from distinct samples or whether the same sample was measured repeatedly
☒	☐	The statistical test(s) used AND whether they are one- or two-sided<br>*Only common tests should be described solely by name; describe more complex techniques in the Methods section.*
☒	☐	A description of all covariates tested
☒	☐	A description of any assumptions or corrections, such as tests of normality and adjustment for multiple comparisons
☒	☐	A full description of the statistical parameters including central tendency (e.g. means) or other basic estimates (e.g. regression coefficient) AND variation (e.g. standard deviation) or associated estimates of uncertainty (e.g. confidence intervals)
☒	☐	For null hypothesis testing, the test statistic (e.g. *F*, *t*, *r*) with confidence intervals, effect sizes, degrees of freedom and *P* value noted<br>*Give P values as exact values whenever suitable.*
☒	☐	For Bayesian analysis, information on the choice of priors and Markov chain Monte Carlo settings
☒	☐	For hierarchical and complex designs, identification of the appropriate level for tests and full reporting of outcomes
☒	☐	Estimates of effect sizes (e.g. Cohen's *d*, Pearson's *r*), indicating how they were calculated

Our web collection on statistics for biologists contains articles on many of the points above.

Software and code

Policy information about availability of computer code

Data collection	The theoretical calculations are performed with Vienna Ab initio Simulation Package.
Data analysis	The data are plotted into Figures with OriginPro 2021 edition.

For manuscripts utilizing custom algorithms or software that are central to the research but not yet described in published literature, software must be made available to editors and reviewers. We strongly encourage code deposition in a community repository (e.g. GitHub). See the Nature Portfolio guidelines for submitting code & software for further information.

Data

Policy information about availability of data

All manuscripts must include a data availability statement. This statement should provide the following information, where applicable:

- Accession codes, unique identifiers, or web links for publicly available datasets
- A description of any restrictions on data availability
- For clinical datasets or third party data, please ensure that the statement adheres to our policy

All data supporting the research in this study are available within the article and supplementary information file. Source data are provided in this paper.

Research involving human participants, their data, or biological material

Policy information about studies with human participants or human data. See also policy information about sex, gender (identity/presentation), and sexual orientation and race, ethnicity and racism.

Reporting on sex and gender	Human research participants and Sex and Gender in research were not included in this study.
Reporting on race, ethnicity, or other socially relevant groupings	Race, ethnicity, or other socially relevant groupings in research were not included in this study.
Population characteristics	Population characteristics were not included in this study.
Recruitment	Recruitment was not included in this study.
Ethics oversight	Ethics oversight was not included in this study.

Note that full information on the approval of the study protocol must also be provided in the manuscript.

Field-specific reporting

Please select the one below that is the best fit for your research. If you are not sure, read the appropriate sections before making your selection.

☐ Life sciences ☐ Behavioural & social sciences ☒ Ecological, evolutionary & environmental sciences

For a reference copy of the document with all sections, see nature.com/documents/nr-reporting-summary-flat.pdf

Ecological, evolutionary & environmental sciences study design

All studies must disclose on these points even when the disclosure is negative.

Study description	Comprehensive mechanistic studies were conducted to unveil the semiconductor-rare concerted proton-coupled electron transfer (CPET) mechanism for C–H bond activation using Zn-In-S photocatalyst under visible light irradiation.
Research sample	The research samples are mainly focused on Zinc-indium-sulfides of with varying stoichiometric ratios.
Sampling strategy	XRD, TEM, DRS, photo-electrochemical measurements were conducted to characterize the materials.
Data collection	X.W. (Xuejiao Wu) performed most of the experiments, analyzed the data, and co-wrote the paper. X.F. performed most of the DFT computations and analyzed the data. S.X. performed some of the experiments and analyzed the experimental data. I.S. performed the qualification and quantification of some products. X.W. (Xiaojian Wen) performed part of the DFT computations. D.V. co-wrote the paper. J.C. guided the computational work, analyzed all computational data, and co-wrote the paper. B.S. designed and guided the study, and co-wrote the paper.
Timing and spatial scale	The data collection started on October 10, 2020, and concluded on March 20, 2024. Most experimental work was conducted at KU Leuven, while the computational work and a minor portion of the experimental work took place at Xiamen University.
Data exclusions	No data were excluded from the analyses.
Reproducibility	Exact numbers for each individual type of experiment are provided in Figure legends and in the Source Data file. All attempts to repeat the experiment were successful.
Randomization	Solutions containing samples were randomly added dropwise onto the Cu grids. The corresponding particles were randomly selected in TEM and HRTEM grids.
Blinding	Not applicable

Did the study involve field work? ☐ Yes ☒ No

Reporting for specific materials, systems and methods

We require information from authors about some types of materials, experimental systems and methods used in many studies. Here, indicate whether each material, system or method listed is relevant to your study. If you are not sure if a list item applies to your research, read the appropriate section before selecting a response.

Materials & experimental systems

n/a	Involved in the study
☒	☐ Antibodies
☒	☐ Eukaryotic cell lines
☒	☐ Palaeontology and archaeology
☒	☐ Animals and other organisms
☒	☐ Clinical data
☒	☐ Dual use research of concern
☒	☐ Plants

Methods

n/a	Involved in the study
☒	☐ ChIP-seq
☒	☐ Flow cytometry
☒	☐ MRI-based neuroimaging

Plants

Seed stocks	Plants in research was not included in this study.
Novel plant genotypes	Plants in research was not included in this study.
Authentication	Plants in research was not included in this study.
